# Supplementary figures and images for: Topological data analysis distinguishes parameter regimes in the Anderson-Chaplain model of angiogenesis
Source: PLoS Comput Biol. 2021 Jun 28;17(6):e1009094. doi: 10.1371/journal.pcbi.1009094 (PMC8270459; doi:10.1371/journal.pcbi.1009094)

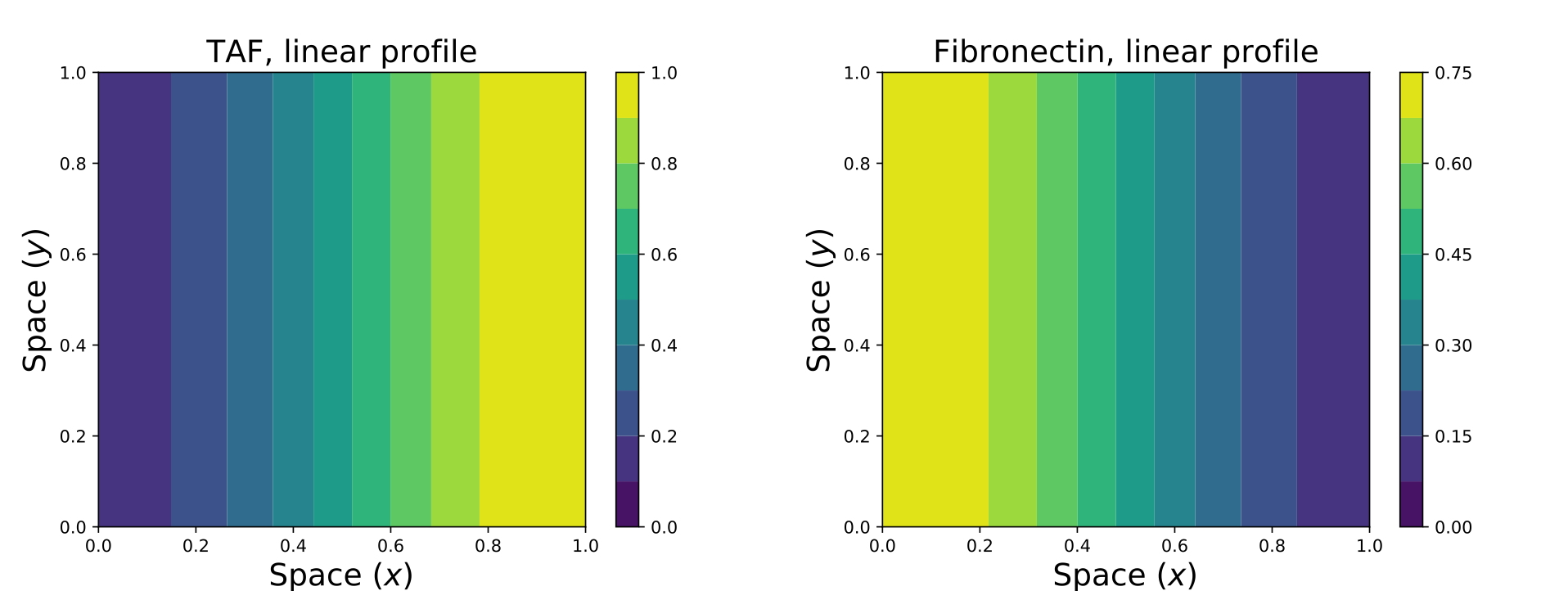

Supplement: S1 Fig — Clustering of the (ρ, χ) parameter space using k-means with k = 5 on the standard descriptor vectors to summarize each simulated vasculature. We considered A) Number of tips over time, C) Number of vessel segments over time, E) Final length of the simulated vasculature, and G) All of the previous descriptor vectors concatenated into one descriptor vector. The five clusters are ordered according to the mean χ value within the cluster. Panels B,D,F,H depict the out of sample confusion matrices for each descriptor vector. (TIF) [file pcbi.1009094.s001.tif]

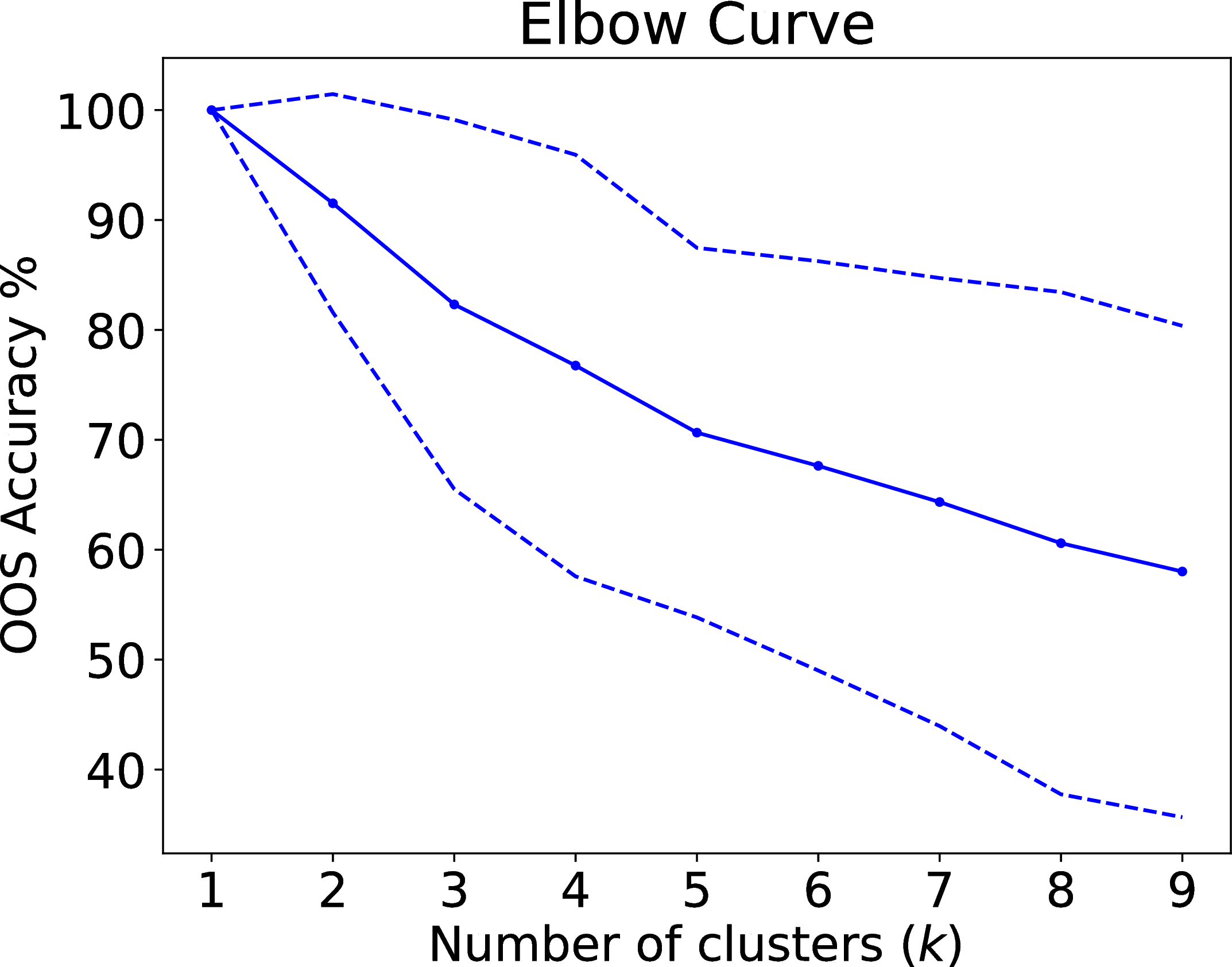

Supplement: S2 Fig — Clustering of the (ρ, χ) parameter space using k-means with k = 5 on individual sweeping plane topological filtrations to summarize each simulated vasculature. The four highest OOS accuracies resulted from the A) PIR1(KLTR), C) PIO0(KLTR), E) PIO1(KRTL), G) and PIO1(KLTR) descriptor vectors. The five clusters are ordered according to the mean χ value within the cluster. Panels B,D,F,H depict the out of sample confusion matrices for each descriptor vector. (TIF) [file pcbi.1009094.s002.tif]

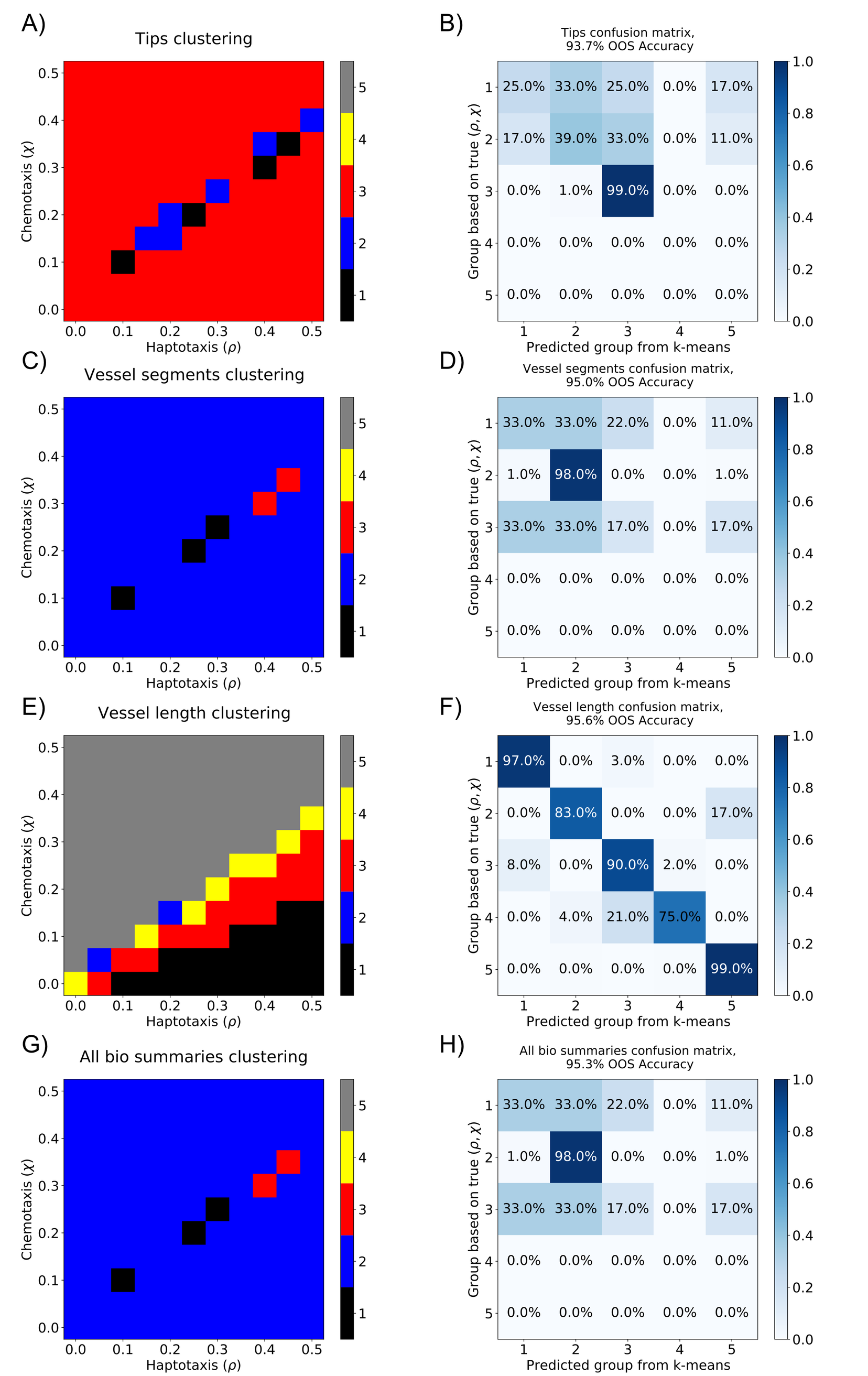

Supplement: S3 Fig — Clustering of the (ρ, χ) parameter space using k-means with k = 5 on double vectors of the sweeping plane topological filtration to summarize each simulated vasculature. The four highest OOS accuracies resulted from the A) PIO0(KLTR) & PIO0(KRTL), C) PIO0(KRTL) & PIR1(KLTR), E) PIO0(KTTB) & PIR1(KLTR), and F) PIO0(KBTT) & PIR1(KLTR) descriptor vectors. The five clusters are ordered according to the mean χ value within the cluster. Panels B,D,F,H depict the out of sample confusion matrices for each descriptor vector. (TIF) [file pcbi.1009094.s003.tif]

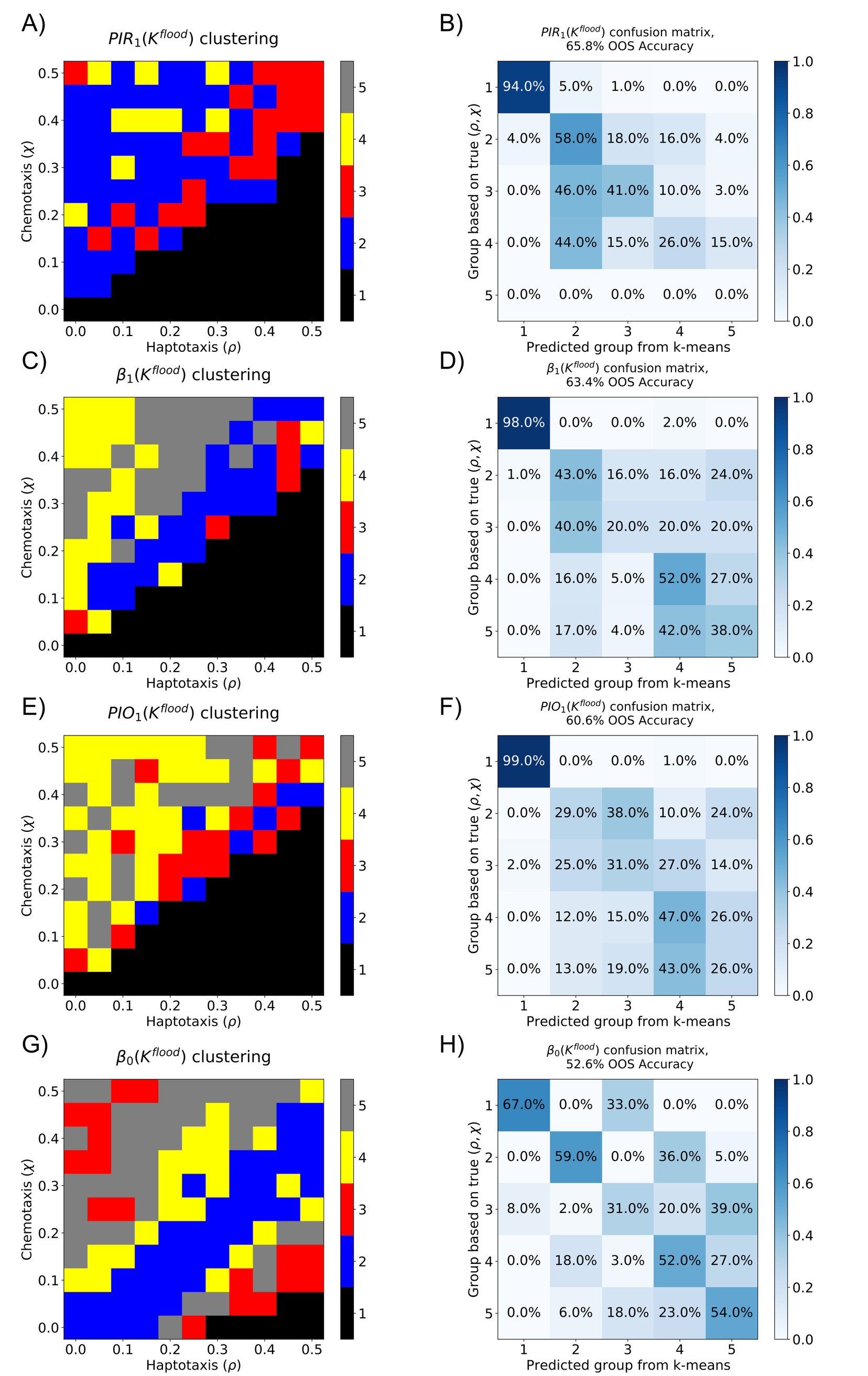

Supplement: S4 Fig — Dimensionality reduction of the PIO0(KLTR) & PIO0(KRTL) double descriptor vector. We reduced the dimensionality of the PIO0(KLTR) & PIO0(KRTL) double descriptor vector to two dimensions using principal components analysis and plot the reduced-dimension descriptor vector for each simulation. The color of each dot denotes the predicted grouping of each simulation from the k-means algorithm. (TIF) [file pcbi.1009094.s004.tif]

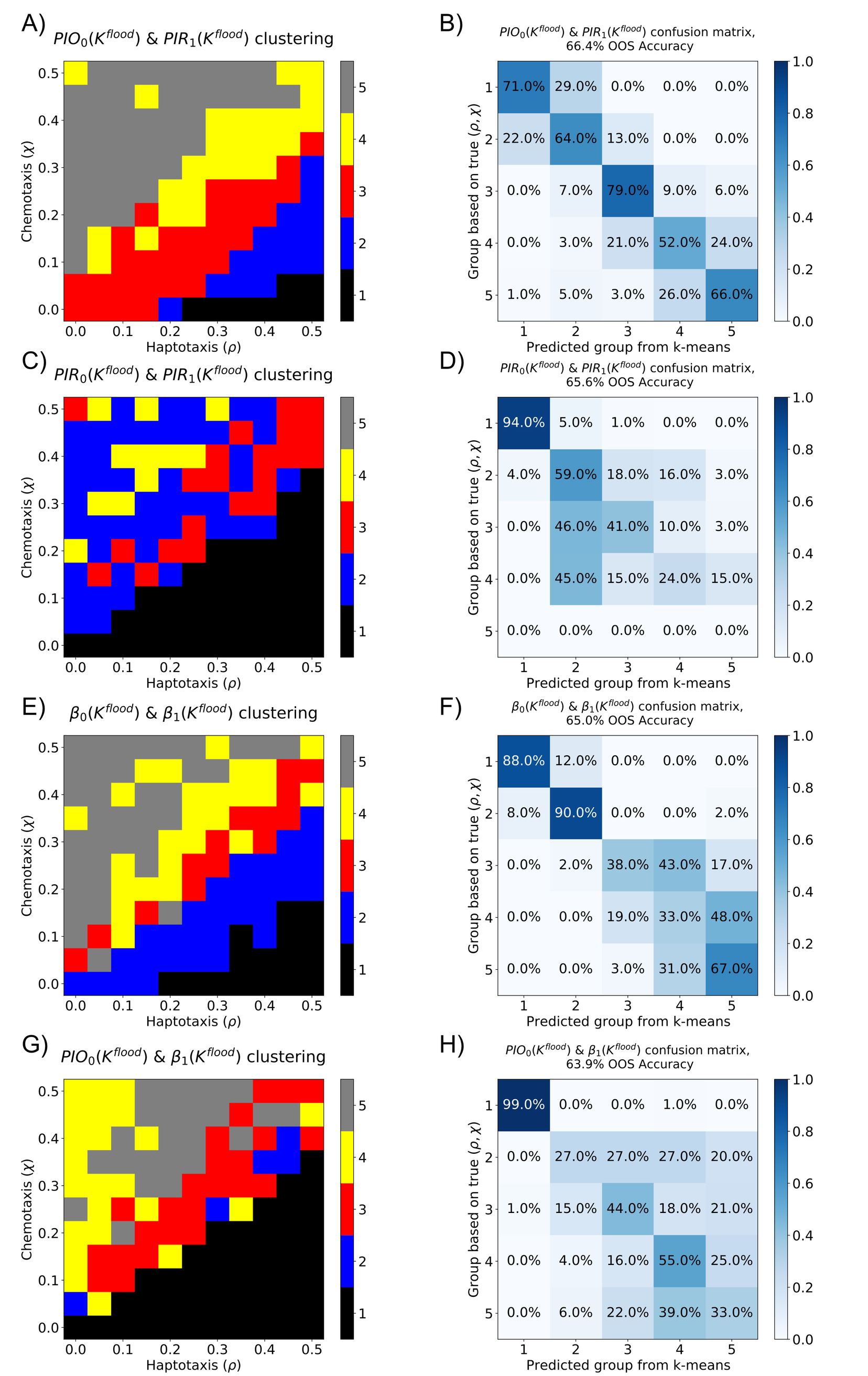

Supplement: S5 Fig — Elbow curve for the OOS Accuracy for the k-means clustering and labeling algorithm over different values of k. We considered all 276 doubles of topological feature vectors and plot the mean OOS accuracy percentage plus or minus two standard deviations for multiple values of k. We propose that the elbow occurs at k = 5. (TIF) [file pcbi.1009094.s005.tif]

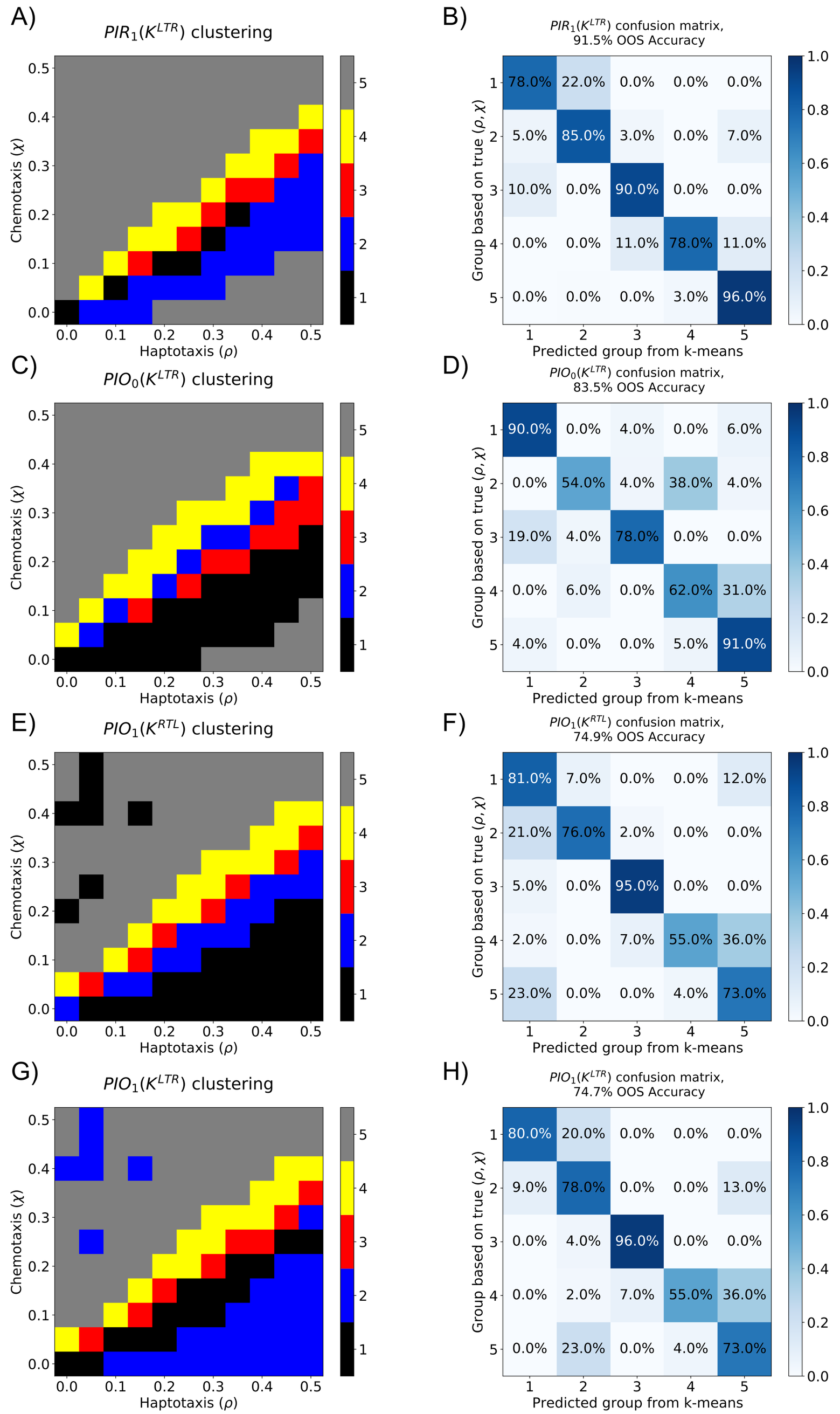

Supplement: S6 Fig — Clustering of the (ρ, χ) parameter space using the PIO0(KLTR) & PIO0(KRTL) double of topological descriptor vectors using the k-means algorithm with A) k = 3, C) k = 4, E) k = 5. The clusters are ordered according to the mean χ value within the cluster. Panels B,D,F) depict the out of sample confusion matrices for each descriptor vectory. (TIF) [file pcbi.1009094.s006.tif]

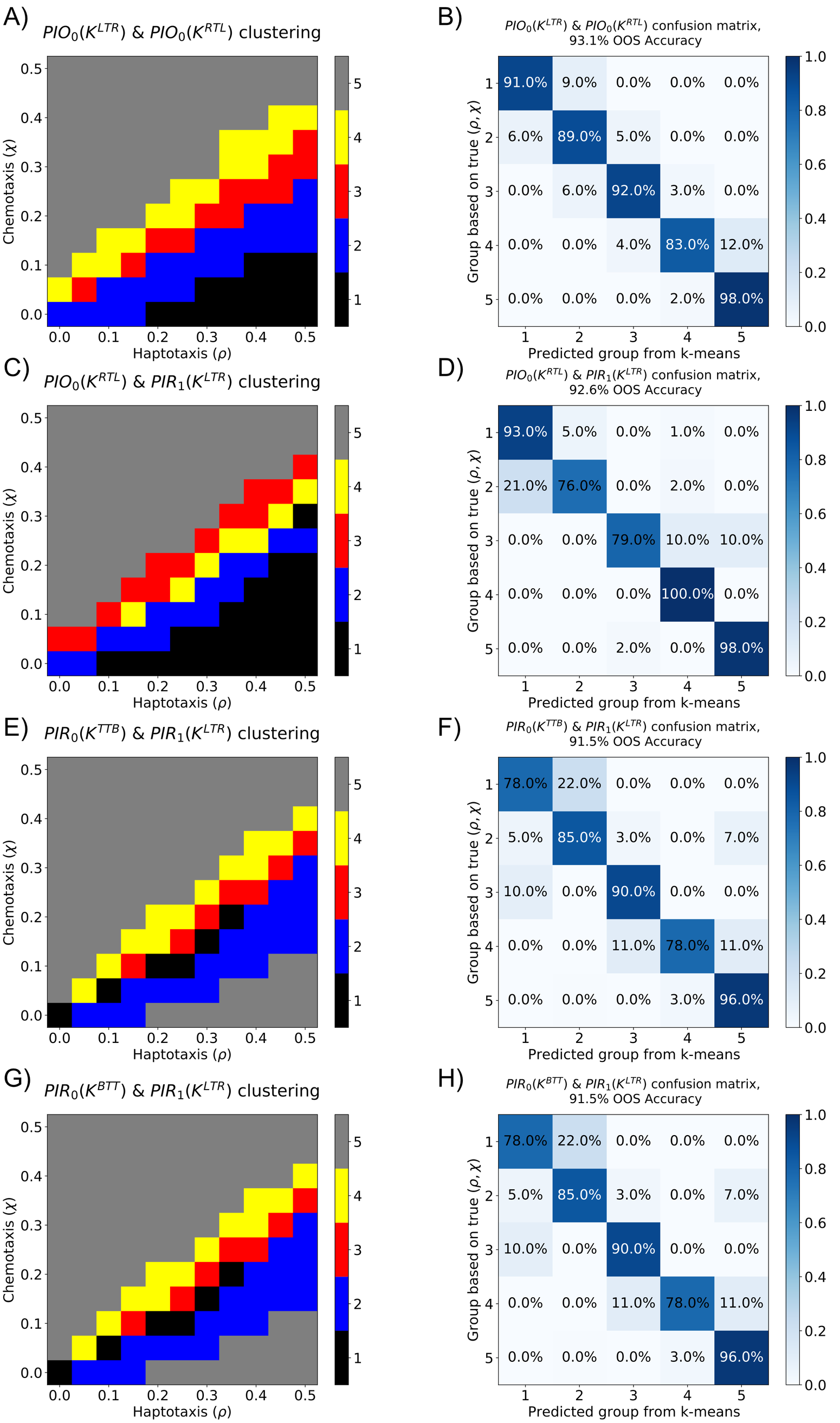

Supplement: S7 Fig — Clustering based on the (ρ, χ) grouping and the time to branch, ψ. We created a data set of blood vessel simulations by considering (ρ, χ) values from groups 1–5 from Fig 13A and letting ψ vary over the 10 values {.1, 0.2, 0.3, …, 1.0}. We simulated the Anderson-Chaplain model ten times at each (ρ, χ, ψ) values to create 500 total simulations. We performed our clustering methodology on this collection of images based off the PIO0(KLTR) & PIO0(KRTL) descriptor vector. (TIF) [file pcbi.1009094.s007.tif]

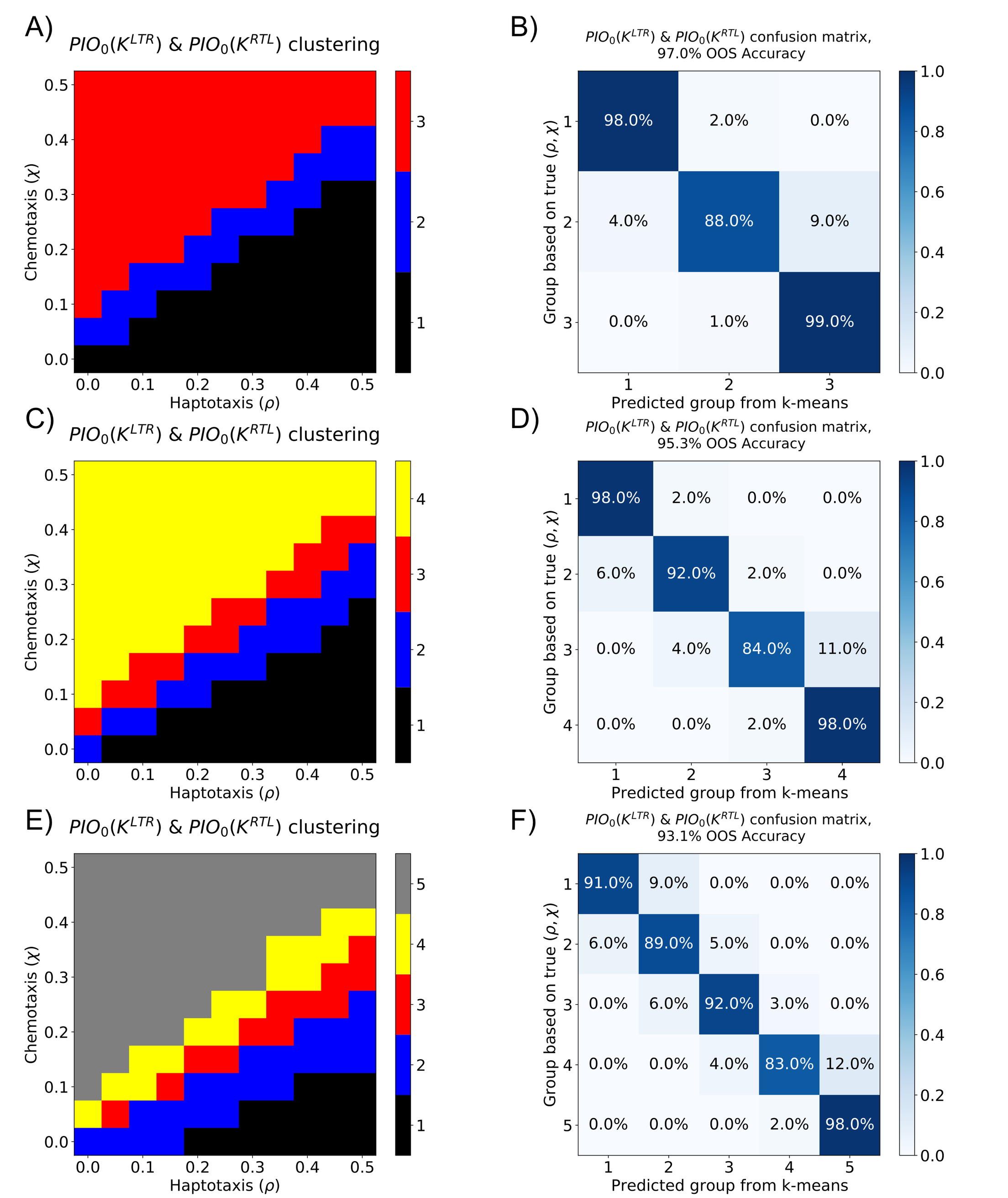

Supplement: S8 Fig — TAF concentration (left) and fibronectin concentration (right). (TIF) [file pcbi.1009094.s008.tif]

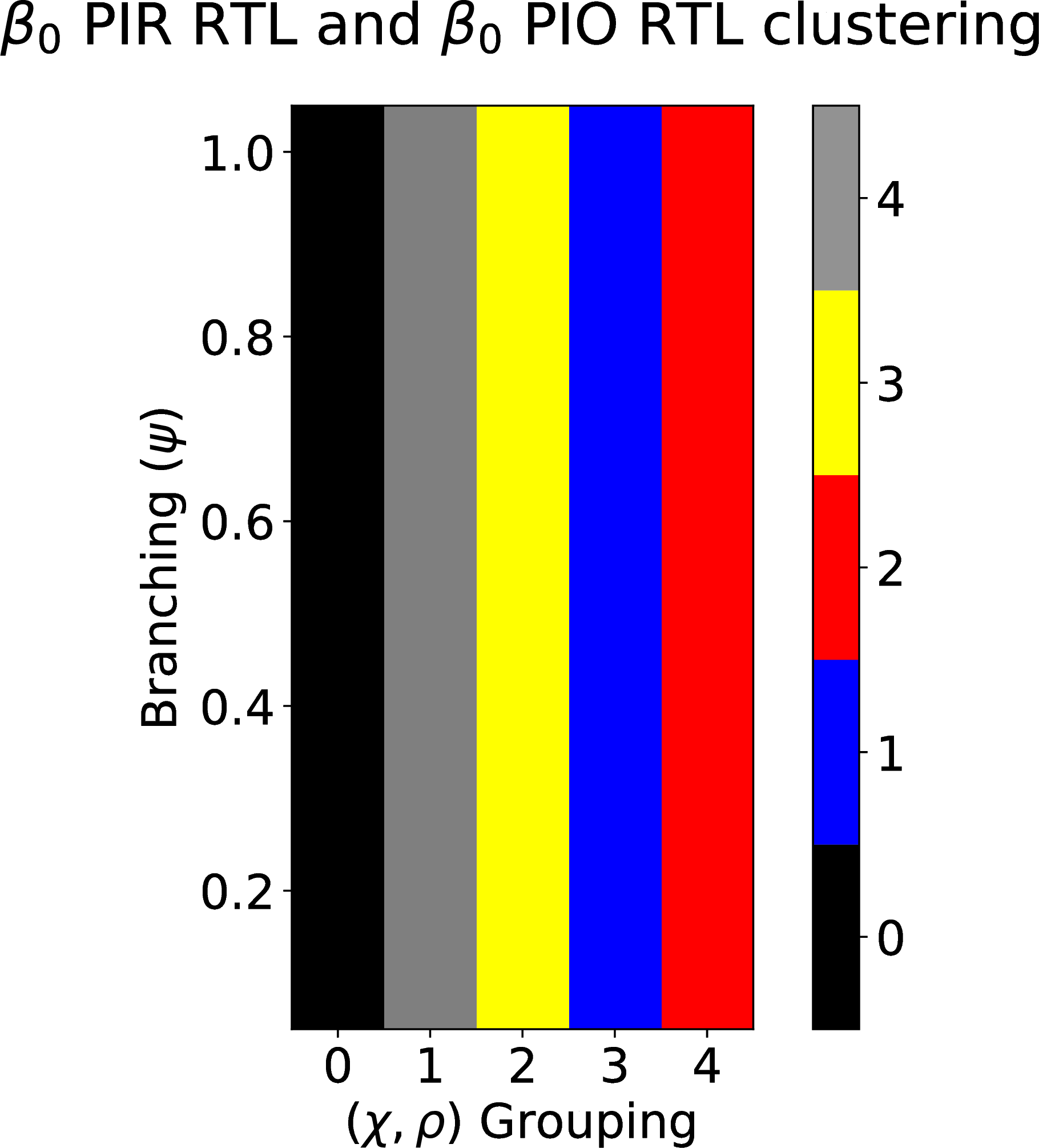

Supplement: S9 Fig — Clustering of the (ρ, χ) parameter space using k-means with k = 5 on individual flooding topological descriptor vectors to summarize each simulated vasculature. The four highest OOS accuracies resulted from the A) PIR1(Kflood), C) β1(Kflood), E) PIO1(Kflood), G) and β0(Kflood) descriptor vectors. The five clusters are ordered according to the mean χ value within the cluster. Panels B,D,F,H depict the out of sample confusion matrices for each descriptor vector. (TIF) [file pcbi.1009094.s009.tif]

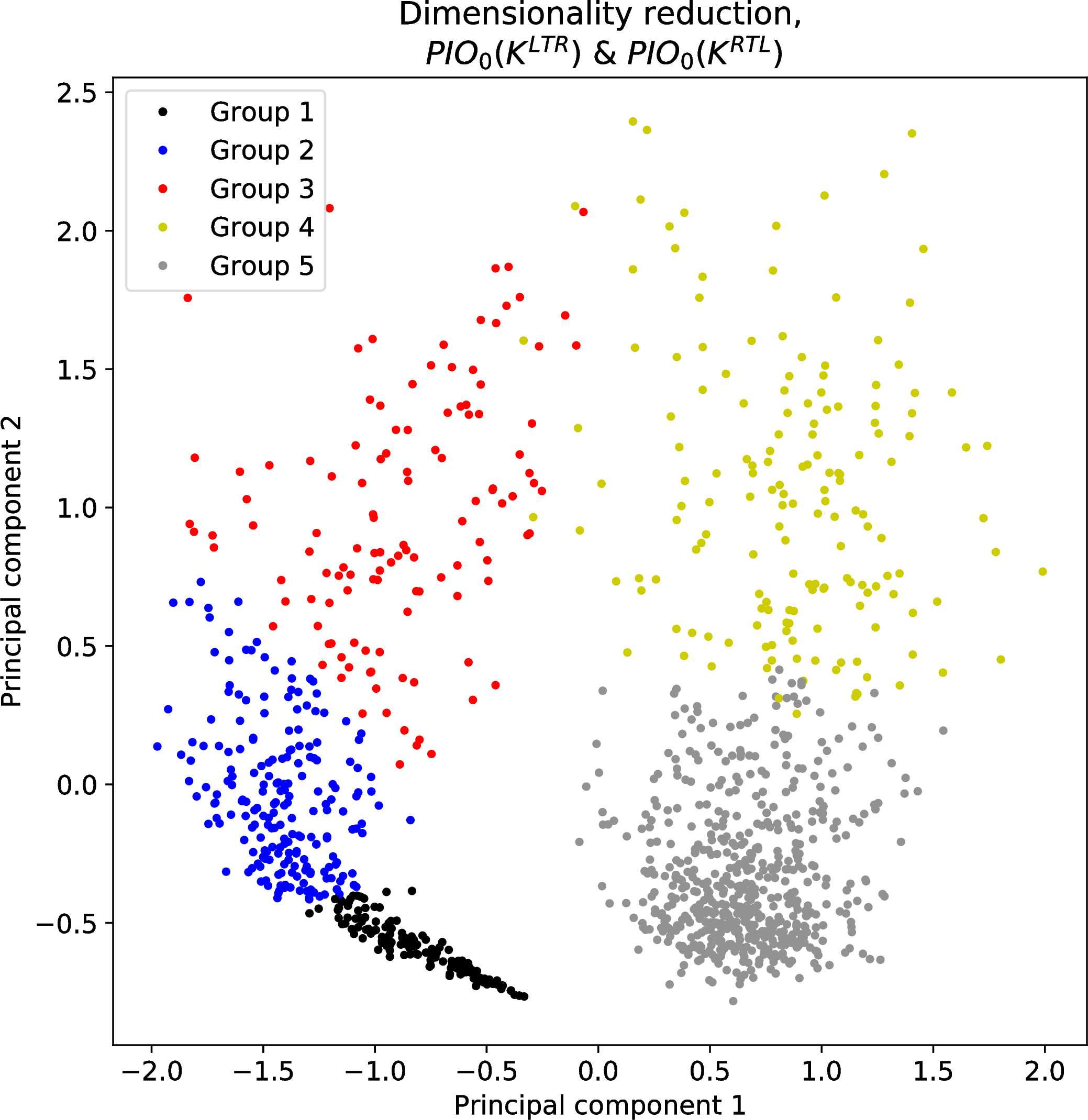

Supplement: S10 Fig — Clustering of the (ρ, χ) parameter space using k-means with k = 5 on double flooding topological descriptor vectors to summarize each simulation. The four highest OOS accuracies resulted from the A) PIO0(Kflood)&PIR1(Kflood), C) PIR0(Kflood)&PIR1(Kflood), E) β0(Kflood)&β1(Kflood), G) and PIO0(Kflood)&β1(Kflood) descriptor vectors. The five clusters are ordered according to the mean χ value within the cluster. Panels B,D,F,H depict the out of sample confusion matrices for each descriptor vector. (TIF) [file pcbi.1009094.s010.tif]
