# Supplementary material for: Topological data analysis distinguishes parameter regimes in the Anderson-Chaplain model of angiogenesis
Source: PLoS Comput Biol. 2021 Jun 28;17(6):e1009094. doi: 10.1371/journal.pcbi.1009094 (PMC8270459; doi:10.1371/journal.pcbi.1009094)
Supplement: S1 Table — List of mechanistic parameters for the Chaplain Anderson ABM from [17]. (PDF) [file pcbi.1009094.s011.pdf]

Topological data analysis distinguishes parameter regimes in the Anderson-Chaplain model of angiogenesis

John T. Nardini[1], Bernadette J. Stolz[2], Kevin B. Flores[1], Heather A. Harrington[2], Helen M. Byrne*[2]

1 Department of Mathematics, North Carolina State University, Raleigh, North Carolina, USA
2 Mathematical Institute, University of Oxford, Oxford, OX2 6GG, UK

* helen.byrne@maths.ox.ac.uk

Parameter	Dimensional value
L length of domain	2 mm
D_n endothelial cell diffusion	$10^{-10}\text{cm}^2/s$
χ_0 chemotactic response	$2600\text{cm}^2/(M \times s)$
ρ_0 haptotactic response	$2600\text{cm}^2/(M \times s)$
f_0 fibronectin concentration	10^{-10} M
D_c TAF diffusion	$2.9 \times 10^{-7}\text{cm}^2/s$
ω Production of fibronectin	1.35×10^{-16}M/s
μ uptake of fibronectin	1×10^{-5}M/s
λ Uptake of TAF	2.7×10^{-6}M/s

S1 Table. Anderson-Chaplain model parameters. List of mechanistic parameters for the Chaplain Anderson ABM from [1].

References

1. Anderson ARA, Chaplain MAJ. Continuous and Discrete Mathematical Models of Tumor-induced Angiogenesis. Bulletin of Mathematical Biology. 1998;60(5):857–899. doi:10.1006/bulm.1998.0042.
