# Supplementary material for: Topological data analysis distinguishes parameter regimes in the Anderson-Chaplain model of angiogenesis
Source: PLoS Comput Biol. 2021 Jun 28;17(6):e1009094. doi: 10.1371/journal.pcbi.1009094 (PMC8270459; doi:10.1371/journal.pcbi.1009094)
Supplement: S2 Table — Baseline nondimensionalized mechanistic parameters for the Chaplain-Anderson Model from [17]. (PDF) [file pcbi.1009094.s012.pdf]

Topological data analysis distinguishes parameter regimes in the Anderson-Chaplain model of angiogenesis

John T. Nardini[1], Bernadette J. Stolz[2], Kevin B. Flores[1], Heather A. Harrington[2], Helen M. Byrne*[2]

1 Department of Mathematics, North Carolina State University, Raleigh, North Carolina, USA
2 Mathematical Institute, University of Oxford, Oxford, OX2 6GG, UK

* helen.byrne@maths.ox.ac.uk

Parameter	baseline value
D	0.00035
χ	0.38
ρ	0.34
β	0.05
γ	0.1
ϵ_1	0.45
ϵ_2	0.45
k	0.75

S2 Table. Anderson-Chaplain model nondimensionalized parameters. Baseline nondimensionalized mechanistic parameters for the Chaplain-Anderson Model from [1].

References

1. Anderson ARA, Chaplain MAJ. Continuous and Discrete Mathematical Models of Tumor-induced Angiogenesis. Bulletin of Mathematical Biology. 1998;60(5):857–899. doi:10.1006/bulm.1998.0042.
