# Supplementary material for: Topological data analysis distinguishes parameter regimes in the Anderson-Chaplain model of angiogenesis
Source: PLoS Comput Biol. 2021 Jun 28;17(6):e1009094. doi: 10.1371/journal.pcbi.1009094 (PMC8270459; doi:10.1371/journal.pcbi.1009094)
Supplement: S4 Table — Out of Sample Accuracy scores for doubles of feature vectors from the flooding filtration using k-means classification with k = 5. (PDF) [file pcbi.1009094.s014.pdf]

Topological data analysis distinguishes parameter regimes in the Anderson-Chaplain model of angiogenesis

John T. Nardini[1], Bernadette J. Stolz[2], Kevin B. Flores[1], Heather A. Harrington[2], Helen M. Byrne*[2]

1 Department of Mathematics, North Carolina State University, Raleigh, North Carolina, USA
2 Mathematical Institute, University of Oxford, Oxford, OX2 6GG, UK

* helen.byrne@maths.ox.ac.uk

Feature	In Sample Accuracy	Out of Sample Accuracy
$\mathrm{PIO}_0(K^{flood})$ & $\mathrm{PIR}_1(K^{flood})$	73.2%	66.4%
$\mathrm{PIR}_0(K^{flood})$ & $\mathrm{PIR}_1(K^{flood})$	71.9%	65.6%
$\mathrm{BC}_0(K^{flood})$ & $\mathrm{BC}_1(K^{flood})$	74.6%	65.0%
$\mathrm{PIO}_0(K^{flood})$ & $\mathrm{BC}_1(K^{flood})$	72.6%	63.9%
$\mathrm{PIR}_1(K^{flood})$ & $\mathrm{BC}_1(K^{flood})$	72.6%	63.4%
$\mathrm{PIR}_0(K^{flood})$ & $\mathrm{BC}_1(K^{flood})$	72.6%	63.4%
$\mathrm{PIO}_1(K^{flood})$ & $\mathrm{BC}_1(K^{flood})$	72.7%	63.4%
$\mathrm{PIO}_0(K^{flood})$ & $\mathrm{PIO}_1(K^{flood})$	70.7%	62.5%
$\mathrm{PIO}_1(K^{flood})$ & $\mathrm{PIR}_0(K^{flood})$	71.2%	60.6%
$\mathrm{PIO}_1(K^{flood})$ & $\mathrm{PIR}_1(K^{flood})$	70.6%	59.8%
$\mathrm{PIO}_0(K^{flood})$ & $\mathrm{BC}_0(K^{flood})$	66.4%	53.4%
$\mathrm{PIO}_1(K^{flood})$ & $\mathrm{BC}_0(K^{flood})$	66.6%	52.1%
$\mathrm{PIO}_0(K^{flood})$ & $\mathrm{PIR}_0(K^{flood})$	62.1%	51.5%
$\mathrm{PIR}_1(K^{flood})$ & $\mathrm{BC}_0(K^{flood})$	66.1%	51.0%
$\mathrm{PIR}_0(K^{flood})$ & $\mathrm{BC}_0(K^{flood})$	66.1%	51.0%

S4 Table. Double flooding clustering. Out of Sample Accuracy scores for doubles of feature vectors from the flooding filtration using k-means classification with $k = 5$.
