# Supplementary material for: Topological data analysis distinguishes parameter regimes in the Anderson-Chaplain model of angiogenesis
Source: PLoS Comput Biol. 2021 Jun 28;17(6):e1009094. doi: 10.1371/journal.pcbi.1009094 (PMC8270459; doi:10.1371/journal.pcbi.1009094)
Supplement: S5 Table — Out of Sample Accuracy scores for individual feature vectors from the sweeping plane filtration using k-means classification with k = 5. (PDF) [file pcbi.1009094.s015.pdf]

Topological data analysis distinguishes parameter regimes in the Anderson-Chaplain model of angiogenesis

John T. Nardini[1], Bernadette J. Stolz[2], Kevin B. Flores[1], Heather A. Harrington[2], Helen M. Byrne*[2]

1 Department of Mathematics, North Carolina State University, Raleigh, North Carolina, USA
2 Mathematical Institute, University of Oxford, Oxford, OX2 6GG, UK

* helen.byrne@maths.ox.ac.uk

Feature	In Sample Accuracy	Out of Sample Accuracy
$\text{PIR}_1(K^{LTR})$	92.3%	91.5%
$\text{PIO}_0(K^{LTR})$	90.1%	83.5%
$\text{PIO}_1(K^{RTL})$	82.4%	74.9%
$\text{PIO}_1(K^{LTR})$	82.6%	74.7%
$\beta_1(K^{LTR})$	82.3%	74.1%
$\text{PIR}_1(K^{RTL})$	78.3%	73.3%
$\text{PIO}_0(K^{RTL})$	82.9%	73.0%
$\beta_1(K^{RTL})$	77.9%	72.7%
$\text{PIR}_0(K^{RTL})$	78.0%	72.7%
$\beta_0(K^{RTL})$	79.0%	70.8%
$\beta_1(K^{TTB})$	74.1%	68.6%
$\beta_0(K^{LTR})$	77.7%	66.1%
$\beta_1(K^{BTT})$	71.4%	65.0%
$\text{PIO}_1(K^{BTT})$	73.3%	64.5%
$\text{PIO}_1(K^{TTB})$	73.9%	64.5%
$\beta_0(K^{BTT})$	72.3%	61.4%
$\text{PIR}_1(K^{BTT})$	69.3%	60.6%
$\text{PIO}_0(K^{TTB})$	68.0%	60.1%
$\text{PIR}_1(K^{TTB})$	70.1%	59.8%
$\text{PIO}_0(K^{BTT})$	67.9%	56.5%
$\text{PIR}_0(K^{LTR})$	67.1%	54.8%
$\beta_0(K^{TTB})$	64.6%	50.1%
$\text{PIR}_0(K^{BTT})$	55.6%	41.6%
$\text{PIR}_0(K^{TTB})$	51.9%	36.6%

S5 Table. Individual plane sweeping clustering. Out of Sample Accuracy scores for individual feature vectors from the sweeping plane filtration using k-means classification with $k = 5$.
