# Supplementary material for: Topological data analysis distinguishes parameter regimes in the Anderson-Chaplain model of angiogenesis
Source: PLoS Comput Biol. 2021 Jun 28;17(6):e1009094. doi: 10.1371/journal.pcbi.1009094 (PMC8270459; doi:10.1371/journal.pcbi.1009094)
Supplement: S1 Appendix — The file contains a more detailed description of implementation of the Anderson-Chaplain Model and clustering results. (PDF) [file pcbi.1009094.s016.pdf]

Topological data analysis distinguishes parameter regimes in the Anderson-Chaplain model of angiogenesis

John T. Nardini[1], Bernadette J. Stolz[2], Kevin B. Flores[1], Heather A. Harrington[2], Helen M. Byrne*[2]

1 Department of Mathematics, North Carolina State University, Raleigh, North Carolina, USA
2 Mathematical Institute, University of Oxford, Oxford, OX2 6GG, UK

* helen.byrne@maths.ox.ac.uk

S1 Appendix

Modeling of Tumor-induced angiogenesis

Model Equations for biological movement and reaction

We use the Anderson-Chaplain model [1] to simulate the formation of blood vessel networks in response to tumor-generated chemical gradients, as depicted in Fig 1. This model describes the spatio-temporal evolution of three dependent variables, namely endothelial tip cells, tumor angiogenic factor (TAF), and fibronectin. Endothelial tip cells emerge from a parent blood vessel and migrate towards the tumor in response to spatial gradients of TAF. In practice, tumors secrete multiple TAFs, including vascular endothelial growth factor (VEGF) and basic fibroblast growth factor (bFGF); in the Anderson-Chaplain model, TAF can be viewed as a generic factor. Tip cells migrate by chemotaxis up spatial gradients and also exhibit a small amount of random diffusion. Fibronectin, a major component of the extracellular matrix, also influences tip cell movement; tip cells move, via haptotaxis, up spatial gradients of fibronectin. For a 2D Cartesian geometry, the evolution of the tip cell density can be represented by the following continuous partial differential equation:

$$\frac{\partial n}{\partial t} = D_n \Delta n - \chi_0 \nabla \cdot (n \nabla c) - \nabla \cdot (\rho_0 n \nabla f) \tag{1}$$

where $n = n(t, x, y)$ denotes tip cell density at time t and spatial position (x, y), $f = f(t, x, y)$ denotes fibronectin concentration, and $c = c(t, x, y)$ denotes TAF concentration. The parameters D, χ, and ρ parameterize tip cell diffusion, chemotaxis, and haptotaxis, respectively.

Fibronectin is assumed to be produced and consumed by tip cells and to remain bound to substrate ECM after its production. The evolution of fibronectin concentration can be represented by the following partial differential equation:

$$\frac{\partial f}{\partial t} = \omega n - \mu n f, \tag{2}$$

where the parameters ω and μ parameterize fibronectin production and consumption, respectively.

TAF is assumed to be secreted by the tumor and to diffuse on a scale faster than the endothelial cell migration. We assume tip cells consume TAF while migrating towards

the tumor. The evolution of TAF concentration can be represented by the following partial differential equation:

$$\frac{\partial c}{\partial t} = D_c \Delta c - \lambda n c, \tag{3}$$

where the parameters D_c and λ parameterize TAF diffusion and consumption by tip cells, respectively. Following [1], we will replace Equation (3) with the simpler partial differential equation:

$$\frac{\partial c}{\partial t} = -\lambda n c. \tag{4}$$

The parameters that appear in the model equations (1)-(2) and (4) are listed in S1 Table, together with a brief description of their biological meaning and typical dimensional values taken from [1].

Nondimensionalization, initial & boundary conditions:

Following [1], we nondimensionalize Eqs. (1)–(2) and (4) as follows. We consider the nondimensionalized variables (with asterisks) given by

$$\begin{aligned}
&t^* = \frac{D_c}{L^2} t, \qquad n^* = \frac{n}{n_0}, \qquad c^* = \frac{c}{c_0}, \qquad \rho^* = \frac{\rho}{\rho_0}, \\
&D^* = \frac{D_n}{D_c}, \qquad \chi^* = \frac{\chi_0 c_0}{D_c}, \qquad \rho^* = \frac{\rho_0 f_0}{D_c}, \\
&\beta^* = \frac{\omega L^2 n_0}{f_0 D_c}, \qquad \gamma^* = \frac{\mu L^2 n_0}{D_c}, \qquad \eta^* = \frac{\lambda L^2 n_0}{D_c}.
\end{aligned} \tag{5}$$

We substitute the variables from Equation (5) into Equations (1)–(2) and (4) and find the nondimensionalized system of equations given by (asterisks dropped for notational convenience)

$$\begin{aligned}
\frac{\partial n}{\partial t} &= D\Delta n - \chi \nabla \cdot (n \nabla c) - \rho \nabla (n \nabla f), \\
\frac{\partial f}{\partial t} &= \beta n - \gamma n f, \\
\frac{\partial c}{\partial t} &= -\eta n c.
\end{aligned} \tag{6}$$

We list the nondimensionalized parameters for Equation in S2 Table.

$$\begin{aligned}
\frac{\partial n}{\partial t} &= D\Delta n - \chi \nabla \cdot (n \nabla c) - \rho \nabla \cdot (n \nabla f) \\
\frac{\partial f}{\partial t} &= \beta n - \gamma n f \\
\frac{\partial c}{\partial t} &= -\eta n c
\end{aligned} \tag{7}$$

The dimensionless parameters used in all simulations (unless otherwise noted) are listed in S2 Table.

We assume that the initial distribution of the TAF c can be described as a one-dimensional profile from a tumor source at $x = 1$ as

$$c(x, y, 0) = e^{-\frac{1-x^2}{\epsilon_1}}, (x, y) \in [0, 1] \times [0, 1]. \tag{8}$$

The initial distribution of the fibronectin concentration f is given by

$$f(x, y, 0) = k e^{\frac{-x^2}{\epsilon_2}}, (x, y) \in [0, 1] \times [0, 1]. \tag{9}$$

Equations (8) and (9) are visually depicted in S7 Fig.

We use no-flux conditions at all boundary points to ensure that the net flux of tip cells into the spatial domain is zero (no boundary conditions are needed for fibronectin or TAF since their governing equations do not contain spatial derivatives).

Stochastic ABM implementation:

We simulate Equation (7) by using a stochastic agent-based model (ABM) to describe tip cell locations over time and the resulting concentrations of fibronectin and TAF. In turn, we create network of angiogenic blood vessels comprised of the tip cells and their previous locations. We begin simulations by creating a lattice for our spatial domain given by $\boldsymbol{x} = i\Delta x, i = 0, ..., N, \Delta x = 1/N$ with $N = 201$. The discretization of the second spatial dimension, y, is defined equivalently. We then initialize ten tip cells locations over time, $n_i^{\text{tip}}(t)$, by setting $n_i^{\text{tip}}(0) = \{(0, 0.1i)\}_{i=1}^{9}$.

In order to simulate Eqs. (1)-(2) and (4), we discretize these equations around each $n_i^{\text{tip}}(t)$ over timestep Δt using the upwind method and form five probabilities governing whether the given tip cell will move left, right, up, down, or stay in place. We then set $n_i^{\text{tip}}(t + \Delta t)$ equal to one of these locations based on these five computed probabilities.

We define the ith vessel segment as

$$n_i^{\text{vessel}} = \bigcup_t n_i^{\text{tip}}(t). \tag{10}$$

We also consider tip cell branching, in which a new tip cell is placed in the ABM domain and creates a new vessel segment. As in [1], we use the following three rules to determine when a tip cell branches and forms a new vessel segment:

1. Only sprouts of age greater than some new threshold value, ψ, may branch. We set $\psi = 0.1$. After a tip cell branches, its "age" is re-set to zero.

2. A tip cell can only branch into neighboring lattice sites that are not occupied by another sprout.

3. The endothelial cell density at $n_i^{\text{tip}}(t)$ must be greater than some threshold level given by
$$n_b = \frac{2.5}{c(n_i^{\text{tip}}(t), t)}. \tag{11}$$
We define the endothelial cell density at the point (x, y) as the number of locations occupied by endothelial cells in the 3×3 grid of points centered at (x, y), divided by nine.

If all three of the above conditions are satisfied, then the tip cell will branch and randomly place the new tip cell into one of its eight adjacent and unoccupied lattice sites.

Resulting vasculature image: From an ABM simulation, we can form the entire vasculature by constructing

$$\boldsymbol{N}_{\text{vasculature}} = \bigcup_i n_i^{\text{sprout}}. \tag{12}$$

We further convert this vasculature to a binary image, $\boldsymbol{N}$, where we set each pixel (i, j) to be

$$\boldsymbol{N}(i,j) = \begin{cases} 1 & \text{if } (i\Delta x, j\Delta y) \in N_{\text{network}} \\ 0 & \text{otherwise} \end{cases}. \tag{13}$$

We present three representative realizations of $\boldsymbol{N}$ in Fig 7 that result from different (ρ, χ) combinations. In these figures, red cells have a values of one to denote a sprout, and white cells have a value of zero to denote the absence of a sprout.

Clustering results

Clustering with standard descriptor vectors: The use of the standard biological descriptor vectors does not lead to biologically interpretable clusterings of the (ρ,χ) parameter space (S1 Fig). The clustering of the parameter space using tips and vessel segment descriptor vectors suggested that the majority of simulations belong to one large cluster, which demonstrates that these descriptor vectors cannot distinguish between simulations that result from visually distinct vascular architectures generated by different model parameter values. The final vasculature length descriptor vector led to four distinct clusters in the parameter space, but almost all (ρ,χ) pairs with $\chi > \rho$ are placed into one large cluster (group 5), suggesting that this descriptor vector cannot distinguish over half of the model simulations from each other. Concatenating all three descriptor vectors into one large descriptor vector led again to one dominant cluster. These descriptor vectors clusterings are robust, however, as each achieves a high OOS accuracy of above 90% (S1 Fig).

Clustering with topological flooding descriptor vectors: We clustered our simulated model data set using the the topological flooding descriptor vectors. Each descriptor vector counts either the number of connected components (β_0) or loops (β_1); performs the flooding filtration; and is summarized using a BC, PIR, or PIO, so we considered $2 \times 1 \times 3 = 6$ separate flooding topological descriptor vectors. Each of these six descriptor vectors are ranked according to their OOS accuracy scores in S3 Table. These clustering regions did not appear biologically interpretable, as many of the groups are not well connected in the (ρ, χ) parameter space (S4 Fig). These flooding descriptor vector clusterings are not robust, as we found a highest OOS accuracy of 65.8% is achieved with the $\text{PIR}_1(K^{flood})$ descriptor vector.

We next considered concatenating doubles of topological flooding vectors as a means to improve robustness. We considered all (6 choose 2) = 15 possible flooding descriptor vectors doubles and ranked these clusterings by the highest OOS accuracies in S4 Table. Doubles of flooding descriptor vectors attain only slightly higher OOS accuracy scores than individual flooding descriptor vectors. The $\text{PIO}_0(K^{flood})$ & $\text{PIR}_1(K^{flood})$ double achieved the highest OOS classification accuracy of 66.4%. Though the clustering regions in (ρ, χ) space for this double flooding descriptor vector did appear biologically interpretable, as the five groups are mostly well connected, aside from Group 4 (S5 Fig).

Clustering with individual topological sweeping plane descriptor vectors: We clustered our simulated model dataset using the the topological sweeping plane descriptor vectors. Each descriptor vector counts either the number of connected components (β_0) or loops (β_1); sweeps the plane in one of four directions (LTR, RTL, TTB, and BTT); and is summarized using a Betti curve (BC), persistence image with ramp weighting (PIR), or persistence image with one weighting (PIO), so we computed $2 \times 4 \times 3 = 24$ sweeping plane descriptor vectors for each model simulation. Each of these 24 summaries were ranked according to their OOS accuracy scores in S5 Table. The four highest OOS accuracy scores resulted from the $\text{PIR}_1(K^{LTR})$ (91.5% OOS accuracy), $\text{PIO}_0(K^{LTR})$ (83.5% OOS accuracy), $\text{PIO}_1(K^{RTL})$ (74.9% OOS accuracy), and $\text{PIO}_1(K^{LTR})$ (74.7% OOS accuracy) descriptor vectors. While these clusterings result in high classification scores, the resulting clusters were determined to not be biologically interpretable. For example, S2 Fig shows that the parameter clusterings that result from either the $\text{PIR}_1(K^{LTR})$ or the $\text{PIO}_0(K^{LTR})$ descriptor vector include both high haptotaxis (*e.g.*, (ρ, χ)=(0.5,0)) and high chemotaxis (*e.g.*, (ρ, χ)=(0,0.5)) parameter combinations in the same cluster (group 5), even though these parameter

combinations lead to markedly different vascular morphologies. The $\mathrm{PIO}_1(K^{RTL})$ and $\mathrm{PIO}_1(K^{LTR})$ clusterings also led to clusterings that include high chemotaxis and high haptotaxis simulations within the same cluster (within groups 1 and 2, respectively).

References

1. Anderson ARA, Chaplain MAJ. Continuous and Discrete Mathematical Models of Tumor-induced Angiogenesis. Bulletin of Mathematical Biology. 1998;60(5):857–899. doi:10.1006/bulm.1998.0042.
