# Supplementary material for: Topological data analysis distinguishes parameter regimes in the Anderson-Chaplain model of angiogenesis
Source: PLoS Comput Biol. 2021 Jun 28;17(6):e1009094. doi: 10.1371/journal.pcbi.1009094 (PMC8270459; doi:10.1371/journal.pcbi.1009094)
Supplement: Attachment — Submitted filename: response_to_reviewers.pdf [file pcbi.1009094.s017.pdf]

Response to Reviewers

Topological data analysis distinguishes parameter regimes in the Anderson-Chaplain model of angiogenesis

John T. Nardini, Bernadette J. Stolz, Kevin B. Flores, Heather A. Harrington, Helen M. Byrne

We thank the three Reviewers for their positive and constructive comments on our manuscript. We have now extensively revised our article in line with their suggestions. We include below a point-by-point response to the comments that were raised by each Reviewer. For clarity, we indicate our responses to the comments, together with the actions we have taken to address these comments. In the revised manuscript (and the responses included below), blue text indicates text that has been added during revision of the manuscript.

Reviewer 1

1. **Comment: Introduction (2nd paragraph): Here the authors outline the A-C angiogenesis model, which appears not to account for any vascular remodelling (vessel compression, collapse, lumen remodelling, etc.) - not as far as more contemporary models do, *e.g.*: DOI:10.1016/j.mvr.2015.02.007, DOI:10.1371/journal.pone.0150296, DOI:10.1371/journal.pcbi.1005259, DOI:10.1098/rsif.2016.0918. Therefore, the authors should justify this simplification in their study. Also, they need to elaborate in the paper how and if this would impact the findings of this work. Along the same lines, the authors should justify why they decided to select investigating the A-C model using a two-dimensional space. They have to elaborate and convince on their decision to do so â mainstream state-of-art mathematical models are 3D; thus, a 2D cancer model study may seem too simplistic nowadays. The authors need to elaborate on this matter and explain again if the findings of their TDA study would be different in the case of a A-C model in 3D.**

   Response:

   Response 1: We thank the reviewer for their comments on the Anderson-Chaplain model of angiogenesis, for highlighting many excellent models of angiogenesis and vascular tumour growth, and for highlighting the differences in complexity between 2D and 3D models of angiogenesis and vascular tumour growth: we completely agree with their comments. At the same time, we would like to stress that the focus of our study is to show how concepts from topological data analysis (TDA) can be used to study mathematical models of angiogenesis, *i.e.*, the period during which a solid tumour transitions from being avascular to vascular. In particular, we are not analysing vessel networks within vascular tumours. We wholeheartedly agree that the Anderson-Chaplain model is highly idealised and neglects many physical processes which

influence angiogenesis. It was chosen as the focus of our study because (i) it provides a minimal description of angiogenesis, (ii) it generates vessel networks which are in good agreement with those observed in vivo, and (iii) it is one of the most highly cited mathematical models of angiogenesis.

Action 1: We have included the following text and citations in the Introduction of the revised manuscript.

> *While more detailed theoretical models of angiogenesis have been developed (see reviews [7, 19, 31, 38, 42], we focus on the Anderson-Chaplain model due to its simplicity and wide adoption in the mathematical biology literature. The methods presented in this work can also be used to study alternative models of angiogenesis, such as the phase-field model presented in [48], the 2D model of early angiogenesis and cell fate specification in [42], the 3D hybrid model presented in [34], and multiscale models of vascular tumour growth, such as those presented in [17, 46, 9, 39]*

Response 2: We acknowledge that a 2D model of angiogenesis is no longer state-of-the-art, however, the focus of our study is to provide a proof-of-concept that TDA can be used to study mathematical models of angiogenesis. Simulations of 3D models of angiogenesis (and vascular tumour growth) and their investigation using TDA can be computationally expensive, and may prohibit exhaustive searches of parameter space, particularly for hybrid, multiscale models that are stochastic. For these reasons, we focused on 2D simulations of angiogenesis in this study. We intend to apply our methods to 3D models of angiogenesis and vascular tumour growth in future work. Indeed, in separate work which is currently under review, we have used TDA to analyse 4D (3D space, 1D time) data extracted from in vivo studies of tumour vessel networks [43].

Action 2: To acknowledge this point, we have incorporated the following text in the "Conclusions and discussion" section:

> *Our future analysis of such state-of-the-art modeling techniques will require us to extend the topological methods presented here for spatio-temporal data that describes how multiple cell types (e.g. tumour cells, immune cells and endothelial cells) interact in 3D. In a preliminary study, we applied these methods to 3D data from in vivo studies of tumour vessel networks, which is near the edge of current computational feasibility [43]. Further work in this direction requires significant computational advancements to scale the TDA pipeline to multiple, more complex models and to analyze their parameter landscapes.*

2. **Comment: Introduction (2nd paragraph) + Discussion: The review articles of theoretical cancer models should include more recent ones, opposed to the ones currently cited [41-44], more specifically the reviewer proposes: DOI:10.1200/CCI.18.00068, DOI:10.1088/1478-3975/ab1a09, DOI:10.1016/ .ymeth.2020.02.010.**

Response: We thank the reviewer for highlighting these excellent recent reviews of tumour modelling, and for giving us the opportunity to cite them in the revised manuscript.

Action 1: We now cite additional, more recent review articles in the Introduction:

> *While more detailed theoretical models have been developed (see reviews [7, 19, 31, 38]), we focus on the Anderson-Chaplain model due to its simplicity and wide adoption in the mathematical biology literature.*

Action 2: We have included additional references in the “Conclusions and discussion” Section:

Many models of angiogenesis have been developed and implemented since the Anderson-Chaplain model, as has been extensively reviewed previously [7, 19, 20, 23, 29, 31, 33, 35, 38].

3. **Comment: Figure 1: This figure is nice despite no clear explanation why/how fibronectin gradients are produced, while also the vascular elements recruited by TAFs that are in turn secreted by tumour cells seem too out of scale compared to the “mother” blood vessel. Minor rework is needed here to further improve the cartoon.**

   Response: We thank the reviewer for their helpful suggestions. We have updated Figure 1 and its caption to improve its clarity.

   Action: In the revised figure, we now clearly indicate the spatial gradients of fibronectin and TAF in the same way. In the caption, we explain briefly how the fibronectin gradient is formed. We depict the new, angiogenic blood vessels as strings of endothelial cells to distinguish from the pre-existing parent vessel.

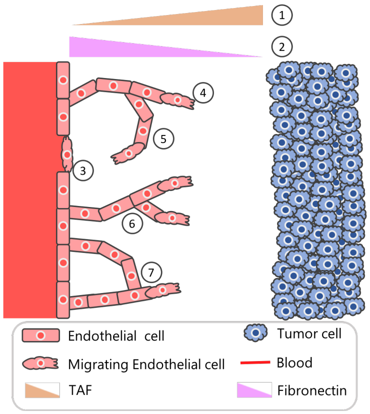

Figure 1: Schematic depicting seven aspects of tumour angiogenesis that are incorporated in the Anderson-Chaplain model of tumour-induced angiogenesis [2]. (1) Distant tumour cells release a range of chemoattractants, including vascular endothelial growth factors and basic fibroblast growth factors that stimulate the formation of new blood vessels. These growth factors are described collectively as a single, generic tumour angiogenesis factor (TAF). (2) Production and consumption of tissue-matrix bound fibronectin by the endothelial cells, creates a spatial gradient of fibronectin across the domain. (3) Endothelial cells sense the TAF and fibronectin gradients and undergo individual cell migration. (4) As endothelial tip cells migrate, via chemotaxis, up spatial gradients of the TAF, stalk cells in the developing vessels are assumed to proliferate, creating what has been termed a “snail trail” of new endothelial cells. (5) Endothelial tip cells also migrate, via haptotaxis, up spatial gradients in fibronectin. (6) Endothelial cells in existing sprouts may initiate the formation of secondary sprouts. (7) If a sprout coincides with an existing vessel, then it is assumed to be annihilated and a new loop is formed; if two sprouts coincide, or anastomose, then both are assumed to be annihilated and a new loop forms.

4. **Comment: Figure 2: Here the authors outline the hierarchy of the proposed pipeline, where the 3 major components (mathematical model -> data analysis -> data clustering) are shown very nicely. However, one may say that box 2 and box 3 concern about data processing and analysis; therefore, there is no clear distinction between the two. Also, there is no connection amongst the 3 boxes that is to say which component from box 1 feeds another component from box 2, and which component from box 2 supplies box 3 or/and box 1, and so on. The illustration needs some rework to render it a true figure showing the "flow" of the processes involved in the proposed pipeline!**

   Response: We agree that the original version of Figure 2 did not show how information flows between the different steps of the pipeline, and thank the reviewer for giving us the opportunity to correct this.

   Action: In the new figure, we have included arrows and modified the caption to describe how the synthetic data produced during step 1 is analysed using both standard descriptors and TDA. Both descriptor vectors are then used as input for the final, data clustering step 3. We believe these new descriptions enable the reader more readily to understand what we mean by "data analysis" and "data clustering" in steps 2 and 3.

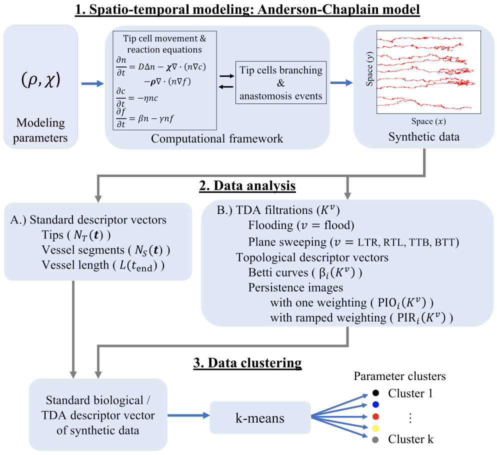

Figure 2: Data generation and analysis pipeline. 1. Spatio-temporal modeling: Anderson-Chaplain model. The Anderson-Chaplain model is simulated for $11 \times 11 = 121$ different values of the haptotaxis and chemotaxis parameters, (ρ, χ). Model outputs are saved as binary images, where pixels labeled 1 (or 0) denote the presence (or absence) of blood vessels. We generate 10 realizations for each of the 121 parameter combinations, leading to 1,210 binary images of simulated vessel networks. 2. Data analysis. We use the binary images from Step 1 to generate standard and topological descriptor vectors. A) Standard descriptor vectors. We compute the number of active tip cells and the number of vessel segments at discrete time points. We also compute the length of each vessel at the final simulation time point [13, 25, 24, 1]. B) Topological descriptor vectors. We construct flooding and sweeping plane filtrations [5] using binary images from the final time point of each simulation. We track the birth and death of topological features (connected components and loops) and summarize the results with Betti curves and persistence images [1]. 3. Data clustering. We perform k-means clustering using either the standard or topological descriptor vectors computed during step 2 from all 1,210 simulations. In this way, we decompose (ρ, χ) parameter space into regions that group vessel networks with similar morphologies.

5. <u>Comment:</u> **Materials Methods: The major direction of tumour angiogenesis data via a topological data analysis of the Anderson-Chaplain model is to interrogate haptotaxis and chemotaxis. However, the papers cited already in this manuscript [47,48] highlight the importance of mechanotaxis in tumour angiogenesis. This is a major simplification which may also have significant impact to the TDA analysis of the tumour networks. The authors need to elaborate on this in the paper, and they are strongly advised to encompass in their study the effect of mechanotaxis.**

   <u>Response:</u> We have included additional text in the conclusion and discussion section which elaborates on the simplifications made in the Anderson-Chaplain model of angiogenesis, which we have used in the present study. In particular, we state clearly that the model neglects mechanotaxis (and many other biophysical processes), and discuss the potential impact that mechanotaxis has on the angiogenesis model and downstream TDA analysis.

   <u>Action:</u> The following text have been added to the "Conclusions and discussion" section:

   > *Even though the Anderson-Chaplain model neglects mechanotaxis, subcellular signalling, and many other biophysical processes, it has been widely adopted due to its ability to generate networks which are in good agreement with those seen in vivo. More complex models have been developed that account for mechanical factors such as mechanotaxis, pressure-driven convective transport of extracellular factors, mechanical stimulation of endothelial cell proliferation, and subcellular signalling pathways that guide cell fate specification of tip and stalk cells [46, 47, 34, 42]. Importantly, these more complex models have been shown to recapitulate experimental observations of time-varying spatial distributions of angiogenic vasculature [46] and features such as asymmetric neovasculature [47] and cell rearrangements and phenotype switching of endothelial stalk and tip cells [21, 10]. In future work we will apply the TDA framework developed here to these, and other, detailed angiogenesis models: since our framework only relies on a segmented vasculature image, this should, in principle, be relatively straightforward. Such analyses will enable us to investigate the effect that processes such as mechanotaxis and subcellular signalling have on the topology of blood vessel networks.*

6. <u>Comment:</u> **Materials Methods: "We generate 10 realizations of the model for each of the 121 parameter combinations, and produce... " It should be justified how this parameter space discretization and why this parameter range was selected. Should the authors have selected a much broader or narrower parameter range for model parameters ârho' and âchi', or even a parameter range that may produce "unrealistic simulations," would this have affected the findings significantly, or not? Please elaborate on this â the authors should also connect their response with the above reviewer comment!**

   <u>Response:</u> We agree that additional discussion is needed about the selection of the parameter range and discretization. We added text in the Materials and Methods section that describes this selection.

   <u>Action:</u> The following text has been added to the Materials and Methods subsection "Data generation":

   > *The parameter bounds of [0,0.5] were chosen to yield a range of vascular architectures that are visibly distinct and recapitulate vascular patterns seen in vivo. The resolution of the parameter mesh (11 discrete values between*

0 and 0.5) was chosen for computational tractability while still being sufficiently detailed to illustrate how TDA methods can detect and quantify differences between simulations generated using similar parameter values that are difficult to distinguish visually.

7. **Comment: Materials Methods: "To facilitate downstream clustering analysis, we require vectors of the same length for all simulations." The authors need to justify this decision. What if the âexperimental data' were not homogeneous and of a different sample size. Would this impact the reliability and accuracy of the proposed pipeline; would the pipeline be able to work in principle? Please explain that in the manuscript.**

   Response: We have added additional text to the Materials and Methods section to explain why all vectors are chosen to be the same length, noting that this requirement is only for the non-TDA related methods and, thus, does not affect any results from the TDA methodology.

   Action 1: The following text has been added to the Material and Methods subsection "Morphologically-motivated standard descriptors and descriptor vectors":

   > *We remark that the requirement for having vectors of the same length for all simulations might not be feasible when dealing with experimental data, which could include heterogeneities in sample size and time point locations. We note further that this requirement only applies to the standard descriptors; for the topological filtrations considered in future sections, only the final segmented vessel image is needed.*

   Action 2: The following text has been added to the "Quantifying vessel shape using topological data analysis" Section:

   > *From the constructed simplicial complexes, one can quantify and visualize the connected components (dimension 0) and loops (dimension 1) of a dataset at different spatial scales. While we compute the N_T and N_S standard descriptor vectors at 50 time points for each model simulation (see Section Morphologically-motivated standard descriptors and descriptor vectors), we compute topological descriptor vectors from the final output binary image.*

8. **Comment: Figure 3: Selection of vessel segments (that would in principle determine the number of vessel segments) appears somehow arbitrary. Please explain how it works in the algorithm. Also, the concept determining the final length of the vessels may be appropriate for a 2D representation of a vasculature. Despite of this, what if one had considered to adopt a different wave-front determination algorithm for the tip vessel propagation "monitoring." What if one had employed in the algorithm multiple wave-fronts / directions of the dashed-line(s). Would the proposed approach work in 3D representation of the tumour vessels? Please elaborate further details of the algorithm in the manuscript.**

   Response:

   Response 1: We agree that our original definition of vessel segments was not clear and have modified this definition in the following text.

Action 1: We have revised the text in two places to clarify our definition for blood vessel segments.

1.) In the Section "Morphologically-motivated standard descriptors and descriptor vectors," we now write:

> *As described in Methods in the appendix, N_S and N_T both increase by one with each branching event, and N_T decreases by one with each anastomosis event...*

2.) In Section "Stochastic ABM implementation" of the appendix, we now write:

> *We define the ith vessel segment as*
>
> $$n_i^{vessel} = \bigcup_t n_i^{tip}(t). \tag{1}$$
>
> *We also consider tip cell branching, in which a new tip cell is placed in the ABM domain, and creates a new vessel segment. As in [2], we use the following three rules to determine when a tip cell branches and forms a new vessel segment.*
>
> - *Only sprouts of age greater than some new threshold value, ψ, may branch. We set $\psi = 0.1$. After a tip cell branches, its "age" is re-set to zero.*
> - *A tip cell can only branch into neighboring lattice sites that are not occupied by another sprout.*
> - *The endothelial cell density at $n_i^{tip}(t)$ must be greater than some threshold level given by*
>
> $$n_b = 2.5c(n_i^{tip}(t), t). \tag{2}$$
>
> *We define the endothelial cell density at the point (x, y) as the number of locations occupied by endothelial cells in the 3×3 grid of points centered at (x, y), divided by nine.*

Response 2: We agree that it would be interesting to determine how tracking the length of the network in multiple directions impacts our analysis, particularly when the studying the remodelling and adaptation of vascular tumour networks. In the present study, we focus on one direction because the fibronectin and TAF gradients establish a clear bias in tip cell movement. In future work, we plan to consider 3D networks that expand in multiple directions, in which the consideration of length in multiple directions will be necessary in order to extract the degree of anisotropy in the evolving blood vessel network. We agree that it would be interesting to apply the standard descriptors to data from three dimensional vessel networks in our study, but consider it beyond the scope of the current article. We note that, in separate work, we have used standard and topological methods to analyse data from 3D biological networks (for details, see: [43]).

Action 2: We have included the following text in the "Conclusions and discussion" Section:

> *Our future analysis of such state-of-the-art modeling techniques will require us to extend the topological methods presented here for spatio-temporal data that describes how multiple cell types (e.g., tumour cells, immune cells and endothelial cells) interact in 3D. In a preliminary study, we applied these methods to 3D data from in vivo*

studies of tumour vessel networks, which is near the edge of current computational feasibility [43]. Further work in this direction requires significant computational advancements to scale the TDA pipeline to multiple, more complex models and to analyze their parameter landscapes.

9. Comment: **Filtrations for simulated vasculature / Figure 4: It is not entirely clear in the methods how the binary image is generated â here from the model produced data (synthetic data) â and how it is associated with the resolution of the simulation space. More precisely, the authors need to explain how the "resolution" of the model generated tumour vasculature relates to the binary image "resolution". Is there any relationship between the two resolutions? For example, let's take two extreme scenarios: (a) in the first one, assume the resolution of the model vasculature (produced using the snail-trail algorithm) is of the order of 1 [unit length] (minimum segment length) whereas the pixel size is 10*10 [unit length]^2; (b) in the second scenario, the resolution of the model vasculature is of the order of 1 [unit length] (minimum segment length) whereas the pixel size is 1*1 [unit length]^2. Can the authors explain how the filtration algorithm will perform â please quantify your results.**

   Response: We simulate the model on a 201x201 lattice, and use the same lattice to generate the binary images. For each binary image, a pixel value of zero or one denotes the presence or absence of an endothelial tip cell at that lattice site, respectively. Since the same 201x201 lattice is used for the simulations and the binary images, we are not downsampling the resolution in our study; we observe the data at the individual cell level. We postpone comparison of the performance of our methods on finer and coarser lattices to future work.

   Action 1: The following text has been added to the "Data generation" Section

   > *... We generate 10 realizations of the model for each of the 121 (ρ, χ) parameter combinations, and produce 1,210 binary images that summarize the different synthetic blood vessel networks. Each simulation is initialized using the same initial conditions, with all other model parameters fixed at the baseline values used in [2], and no-flux conditions imposed on the domain boundary. For consistency with [2], all simulations are computed on lattices of size 201×201. Each simulation continues until either an endothelial tip cell crosses the line $x = 0.95$ (in dimensionless spatial units) or the maximum simulation time, $t = 20$ (in dimensionless time units), is exceeded. The final model output is a 201×201 binary image in which nonzero (zero) pixels denote the presence (absence) of an endothelial cell at that lattice site.*

   Action 2: The following text has been added to "Conclusions and discussion" Section:

   > *Our results using persistence images with unit weightings, rather than ramped weightings, also suggest that short persistence features may be informative for distinguishing vessel morphologies. The spatial resolution of the output binary images that we used for our analyses was the same as that used to generate the simulations, enabling us to distinguish the presence and absence of individual endothelial cells at each lattice site. In future work, we will consider finer and coarser binary images to investigate how the performance of the methods depends on their spatial resolution.*

10. <u>**Comment:**</u> **The authors need to comment also about the efficiency of the "sweeping plane filtration" and "flooding filtration" algorithms when applied to 3D data (*i.e.*, image stacks of MR scans, see review DOI:10.1018/nrclinonc.2014.126).**

    <u>**Response:**</u> The reviewer raises an excellent point about the practical considerations of this work. Determining the efficiency of these methods will be particularly important for extending our analysis to 3D data. Persistent homology is an $O(n^3)$ process, where n is the number of simplices in the simplicial complex. We postpone further analysis of the efficiency of our plane sweeping and flooding techniques for future work.

    <u>Action:</u> We have included the following text in the "Conclusions and discussion" section:

    > *Our future analysis of such state-of-the-art modeling techniques will require us to extend the topological methods presented here for spatio-temporal data that describes how multiple cell types (*e.g., *tumour cells, immune cells and endothelial cells) interact in 3D. In a preliminary study, we applied these methods to 3D data from in vivo studies of tumour vessel networks, which is near the edge of current computational feasibility [43]. Further work in this direction requires significant computational advancements to scale the TDA pipeline to multiple, more complex models and to analyze their parameter landscapes.*

11. <u>**Comment:**</u> **The following statement is not entirely clear to the reviewer, please consider rephrasing it. "On each subsequent step, we iterate through each nonzero pixel from the previous binary image and manually set every pixel in its Moore neighborhood ..."**

    <u>**Response:**</u> We have rephrased this text and trust that the meaning is now clear.

    <u>Action:</u> We have rephrased the text in the "Flooding filtration" section:

    > *On the first step, we input the output binary image from a model simulation. In the binary image, pixels with value one denote the presence of an endothelial cell and pixels with value zero denote the absence of endothelial cells. To create the second binary image, we identify all pixels of value one in the initial binary image, and assign all pixels in their Moore neighborhood (as shown in Fig 4B) to value one. We repeat this process, generating a sequence of binary images, until a binary image with only pixels of value one is created.*

12. <u>**Comment:**</u> **"From the corresponding sequence of binary images, we construct a filtered simplicial complex $K^{flood} = \{K_1, K_2, ..., K_{end}\}$." To the reviewer's understanding, end filtration will result into a simplicial complex that covers the entire "image". Can the authors explain why this is needed? Is this right?**

    <u>**Response:**</u> The reviewer's understanding is correct that the end filtration results in a simplicial complex that covers the entire image. As discussed in [11], a filtration of a simplicial complex K is a nested sequence of subcomplexes that starts with the empty complex and ends with the complete complex:

    $$\emptyset = K_0 \subseteq K_1 \subseteq \ldots \subseteq K_{end} = K.$$

In our application, we choose the complete complex K to be the covered image. We chose to only include the first 25 flooding steps in this filtration, however (as depicted in Figure 11), since it results in a β_0 value equal to 1 (only a single connected component) and β_1 value equal to zero (no loops) after K_{end}=25 filtration steps. We note that choosing any larger value of K_{end}, *e.g.*, $K_{end} = 26$, would just result in additional "ones" for β_0 and "zeros" for β_1 being appended to the end of the respective Betti curves, which is non-informative. Choosing any smaller value of K_{end}, *e.g.*, K_{end}=24, would not have resulted in a simplicial complex covering the entire image and filling in all holes for every simulation, and thus the β_0 and β_1 curves for some simulations might not yet have attained values of 1 and 0, respectively. Thus, choosing K_{end}=25, when the Betti curves for all simulations attain β_0=1 and β_1=0, is standard practice in the TDA literature and ensures the maximum amount of topological information is encoded in the betti curves.

Action: To clarify the choice of K_{end}=25, we added the following text to the "Flooding filtration" paragraph in the "Filtrations for simulated vasculature" subsection of the "Quantifying vessel shape using topological data analysis" section:

> *We chose $K_{end} = 25$ so that the Betti curves for all simulations attain $\beta_0 = 1$ and $\beta_1 = 0$ at the final filtration step, thus ensuring that the maximum amount of topological information is encoded in the Betti curves.*

13. **Comment: Pages 8-9: "Intuitively, a topological feature is born in filtration step ... with another component or when a loop is covered by 2-simplices." It is self-evident that the homology group for when a feature is generated / born (tip vessel, branch, anastomosis, etc.) and for when a feature ceases to exist (*e.g.*, tip transforms into an anastomosis). Nonetheless, and in view of the modelling simplification that no vascular remodelling / vessel compression or collapse / vessel intussusception is encompassed in the A-C model, then how a branch (or even a tip vessel) is regressed, or how an anastomosis is pruned in the vascular network.**

Response: We believe that there is some confusion regarding when the model and TDA computations are being performed. We first run each model simulation in full; generating these simulations involves actions such as tip cell migration, branching, and anastomosis but not vascular remodelling, compression, or collapse as these processes are neglected in Anderson and Chaplain's highly idealised model of angiogenesis. When a simulation is complete, we associate, with the vessel network at the final point, a binary image, in which a nonzero entry indicates the presence of an endothelial cell at that pixel, and a zero entry indicates no endothelial cells at that pixel. We only perform our TDA summaries (plane sweeping and flooding) on this binary image (*i.e.*, after model simulation).

Because tip cells are predominantly moving from left to right (due to the TAF gradient), the plane sweeping approach can identify the locations of branching and anastomosis events as follows. An anastomosis event occurs when two vessel segments join together. For the left-to-right (LTR) sweeping plane filtration, when the sweeping plane is located to the left of the anastomosis site, the two vessel segments are represented by two separate connected components. Once the plane reaches the anastomosis location, however, the two segments form a single connected component. Thus, the LTR plane sweeping filtration tends to decrease by one when it reaches the locations of anastamosis events. In a similar fashion, the right-to-left (RTL) plane sweeping filtration decreases by one when it reaches the locations of branching events.

Action: The following text has been included in the "Materials and Methods" section:

> *We compute three so-called standard descriptor vectors to summarize each simulation: two are computed at 50 time steps and the third is computed at the final time step. We also compute 30 topological descriptor vectors.*

The following text has been included in the "Persistent homology" section:

> *In our case, the scale parameter corresponds to the spatial location in the sweeping plane filtration and to the number of flooding steps in the flooding filtration. Note that we compute these filtrations on simulated blood vessel network images at the final time step of a model simulation...*

14. **Comment: Topological descriptor vectors from PH: The following statement may need some further justification why this step was considered in the persistent homology algorithm. "In a first step the coordinates (b; d - b) are blurred by a Gaussian, with standard deviation sigma, that is centered about each birth-persistence point."**

    Response: This step was included as per the original study of persistence images [1]. Gaussian blurring is performed to account for uncertainty associated with where the topological feature may be born and die.

    Action: We have added the following text in in the "Persistence images" section to clarify the rationale for including Gaussian blurring:

    > *A persistence image [1] uses as input the birth-death pairs given by PH and converts the set of (birth b, persistence $(d-b)$) coordinates into a vector, a format which is suitable for machine learning and other classification tasks. Following the standard definition of a persistence image, the coordinates $(b, d-b)$ are blurred by a Gaussian, with standard deviation σ, that is centered about each birth-persistence point, which accounts for uncertainty [1].*

15. **Comment: Simulation Clustering: "Specifically, 7 of the 10 descriptor vectors from each of the 121 (rho, chi) parameter combinations.." were these 7 descriptor vectors begin selected randomly following a specific pattern (uniform, normal distribution)?**

    Response: We have edited this sentence to specify that the random selection was uniform, which is standard practice in machine learning when deriving a validation set.

    Action: The following text has been edited in the Materials and Methods subsection "Simulation Clustering":

    > *Specifically, 7 of the 10 descriptor vectors from each of the 121 (ρ, χ) parameter combinations are randomly chosen, with uniform sampling, and placed into a training set (847 simulations); the remaining 3 descriptor vectors from each (ρ, χ) combination are placed into a testing set (363 simulations).*

16. <u>**Comment:**</u> **Results / Haptotaxis and chemotaxis alter vessel morphology: As covered in the recent reviews (DOI:10.1007/s11831-015-9156-x, DOI:10.1016/j.ymeth.2020.02.010 - they should be cited here), angiogenesis models have already highlighted the importance of chemotaxis and haptotaxis in tumour-induced angiogenesis, whereas in these two modelling papers (DOI: 10.1371/journal.pcbi.1005259) and (DOI:10.1098/rsif.2018.0415) it has been demonstrated the importance of mechanotaxis and interstitial fluid flow in vascular development in the tumour microenvironment. Therefore, the contribution of this paragraph is not entirely clear to the reviewer. It should be enhanced with the analysis suggested in the comment 5 above.**

    <u>**Response:**</u> We thank the reviewer for highlighting the importance of mechanotaxis and interstitial fluid flow on vascular growth and remodelling within vascular tumours. We agree that these processes contribute to the growth and composition of vascular tumours, as do many other biological factors (*e.g.*, subcellular signalling pathways, such as VEGF/delta-notch, haematocrit splitting rules), as have been investigated in the mathematical/computational modelling literature. We reiterate, however, that this paper is not concerned with modelling vascular tumour growth: it is concerned with modelling angiogenesis, *i.e.*, the formation of blood vessels that occurs before a tumour is perfused. We purposely chose a highly idealised model of angiogenesis, which lacks many biological details, to illustrate how TDA can be used to analyse simulated vascular networks. We chose the Anderson-Chaplain model because it is arguably the most highly cited discrete model of angiogenesis in the literature. Thus, we agree wholeheartedly with the reviewer that the model is extremely simplistic. At the same time, however, it generates networks which are in good agreement with those seen in vivo. In future work, we aim to use the topological (and standard) descriptor vectors first to compare different models of angiogenesis, and then to investigate and compare different multiscale models of vascular tumour growth. Consequently we do not consider it appropriate or necessary to include the suggested references at this point in the text, although they are cited at several points throughout the manuscript.

Reviewer 2

1. <u>**Comment:**</u> **The authors frequently make reference to "biologically interpretable / informative / meaningful" clusters. However the definition of "biologically meaningful" is not clear to me; I would guess it refers to clusters that have similar biological properties, but this is purely my inferred meaning. I think the authors should define this up front with a clear statement on what constitutes a "biologically meaningful cluster". Furthermore, there's a difference between "meaningful" and "interpretable"; the latter term has a quite specific meaning in machine learning that is not necessarily equivalent to meaning (an algorithm can be non-interpretable but still produce meaningful predictions).**

   <u>**Response:**</u> We agree with the reviewer that we should have stated more clearly what constitutes a "biologically meaningful cluster." By "biologically interpretable clusterings" we mean that we can identify similarities between simulations in a particular cluster as well as differences between simulations from different clusters. In this specific case study, we propose that the clusters that result from the topological plane sweeping descriptor vectors are biologically interpretable because simulations from the same cluster arise from similar regions in (ρ, χ) parameter space. For example, Fig. 12A exhibits biologically interpretable clusterings, as all simulations in group 1 have large values of haptotaxis and much lower values of chemotaxis (*i.e.*, $\rho >> \chi$). The ρ/χ ratio decreases as we then consider simulations inside groups 2, 3, 4, and finally 5.

   <u>Action 1:</u> We have revised the text in the Introduction to clarify this point:

   > *Our main objectives are to identify descriptor vectors that group model simulations into biologically interpretable clusters. By biologically interpretable, we mean that simulations within a cluster arise from similar parameter values and that simulations from different clusters arise from different parameter values.*
   >
   > *We use two topological approaches, the sweeping plane and flooding filtrations, to construct simplicial complexes from binary image data generated from simulations of the Anderson-Chaplain model. We show that PH of the sweeping plane filtration and its subsequent vectorization provides a descriptor vector which is interpretable for the model parameters governing chemotaxis and haptotaxis. We show, by comparison with existing, morphologically-motivated descriptor vectors, that this topological approach leads to more biologically interpretable clusters that stratify the haptotaxis-chemotaxis parameter space. Furthermore, the clusters generated from the sweeping plane filtration are robust and generalize well to unseen model simulations.*

   <u>Response 2:</u> We agree that we mis-used the phrase "biologically meaningful" at several places in the original manuscript.

   <u>Action 2:</u> For consistency, we have replaced the phrase "biologically meaningful" by "biologically interpretable" throughout the revised manuscript.

2. <u>**Comment:**</u> **Have the authors considered alternative standard descriptors from the literature? For example, δ_{max}, the maximum distance in the tissue from the nearest blood vessel, and λ, a measure of the shape of**

the spaces between vessels, used by https://www.pnas.org/content/108/5/1799. Or even simply tortuosity (which the authors themselves reference on page 13).

Response: We acknowledge that many other standard descriptors could have been used in our study, including those listed by the reviewer. The focus of our study, however, is to highlight how concepts from persistent homology can be used to analyze a collection of simulated vessel networks, rather than to provide an exhaustive analysis of standard descriptors of angiogenesis. We have chosen to consider length, the number of vessel segments, and the number of active tip cells due to their wide use in the literature.

Action: We have added the following text to the "Morphologically-motivated standard descriptors and descriptor vectors" section :

> *Morphologically-motivated standard descriptors are used to summarize physical characteristics of each model simulation. Commonly-used descriptors include inter-vessel spacing, the number of branch points, the total area (volume) spanned by the network for 2-dimensional (3-dimensional) simulations, the shape of such loops in 2D (voids in 3D), and tortuosity [3, 13, 22, 25, 24, 34]. We choose to use the following descriptors due to their prevalence in the literature: the number of vessel segments (N_S), the number of endothelial tip cells (N_T), and the final length of the longest vessel (L) [4, 13, 25, 24].*

3. **Comment: Section "Filtrations for simulated vasculature". The results section raises questions regarding the two representations presented here (sweeping plane and flooding). Given that they produce quite different results, I think some reference should be made to previous applications of each encoding; in particular what problems they were developed to solve, as this may shed light on why sweeping plane performs better than flooding.**

   Response: The previous applications of these two filtrations are limited. The plane sweeping technique was originally considered in [5] to analyse brain artery data. Flooding filtrations have been used in [16] to distinguish retinas from healthy patients and patients with diabetic retinopathy. While TDA has increased in popularity over the past 15 years, researchers have only recently realized the need for application-specific filtrations [43]. We chose to employ the flooding and plane sweeping filtrations after much thought on what an informative filtration may look like for binary images of simulated blood vessel networks. Intuitively, the flooding filtration tracks how topological features change as the vessel segments thicken over each filtration step. The plane sweeping filtration tracks how segments merge and bifurcate as we sweep across the network in different directions.

   Action: We have added references to the following text in section "Filtrations for simulated vasculature":

   > *There are different ways to study vascular data at multiple scales [5, 16, 8, 43]. ... A challenge for researchers is to determine informative filtrations for specific applications [44], such as blood vessel development in our study. Here, the input data is the binary image from the final timepoint of the Anderson-Chaplain model, which we call N (See Eq (13) in the **Appendix**). We construct sequences of binary images that correspond to different filtered simplicial complexes: a sweeping plane filtration [5] and a flooding filtration [16]...*

4. **Comment: Section "Simulation clustering". The distance metric used by the k-means algorithm is not explained; I assume it's Euclidean, but the authors should state this clearly.**

   Response: We have edited the Material and Methods subsection "Simulation Clustering" to clarify that Euclidean distance is used.

   Action: Text in Material and Methods subsection "Simulation Clustering" has been revised as follows:

   > *We use all 363 testing simulations when computing OOS accuracy and use the* `KMeans` *command from scikit-learn (version 0.22.1), which uses Lloyd's algorithm for Euclidean space, for training and prediction.*

5. **Comment: Figs 10, 11. I find it difficult to compare these figures because the Betti curves are presented on different axes (space in Fig 10, step in Fig 11). It would help if the figures were more directly comparable, or even on the same plot.**

   Response: Because of the way in which we have applied the two filtrations, it does not make sense to plot the figures on the same axis. The plane sweeping approach depends on the position of the plane, whereas the flooding approach depends on the number of flooding steps that have been undertaken. The focus of figures 10 and 11 is not directly to compare the two filtrations, but instead to highlight how each of them can be used to distinguish the three simulations presented in Figure 7.

   Action: The following text has been added to the section "Quantification of blood vessel architecture data":

   > *We next illustrate how we use the plane sweeping and flooding filtrations to analyse the chemotaxis-driven, haptotaxis-driven, and chemotaxis & haptotaxis-driven simulations (from Fig 7). We computed Betti curves, $\beta_0(K^v)$ and $\beta_1(K^v)$, of the simulated data with sweeping plane filtrations for $v = \{LTR, RTL, TTB, BTT\}$ (see Fig 10) and a flooding filtration $v = \{flood\}$ (see Fig 11). These filtrations are constructed to provide complementary but not directly comparable information; roughly, the sweeping plane gives an indication of the network in the (x-y) plane whereas the flooding filtration also focusses on vessel density (see earlier description of filtration construction). We could interpret the PH of the sweeping plane filtration direction as follows: the blood vessels grow primarily from left to right, so the LTR and RTL filtrations primarily identify dynamic changes in branching and anastomosis of vessels.*

6. **Comment: Section "Haptotaxis and chemotaxis alter vessel morphology". The respective roles of hapto- and chemotaxis have been investigated before, it's not entirely clear whether the authors are reporting them as new or just including them for context. I would guess the latter, but I think clarification is needed.**

   Response: We agree that this sort of investigation has been performed previously, but we include it in our study for consistency.

   Action: We added the following text in the "Haptotaxis and chemotaxis alter vessel morphology" Section:

We investigated the influence of haptotaxis and chemotaxis by varying the parameters ρ and χ. We note that similar parameter sensitivity analyses have been performed previously [2, 19]; we reproduce this analysis as the basis for our data analysis.

7. **Comment: Section "Sweeping plane descriptor vectors cluster simulations by parameter values". There are two results here that are not explained: i) sweeping plane can produce biologically meaningful clusters, while flooding cannot; ii) double descriptor vectors created by sweeping plane produce biologically meaningful and robust clusters. I think the authors need to provide explanations, and in particular comment on why the two encodings produce such different results.**

   Response: We agree that the results could have been explained more clearly, and apologise for any confusion. A challenge for the application of TDA is to determine how to properly interpret its output. This is commonly done by trial and error, and then one seeks to understand why particular filtrations perform well. We have found that the plane sweeping approach robustly stratifies the (χ, ρ) parameter space into biologically interpretable clusterings. We suggest that this may be because the way in which we create the LTR and RTL filtrations (moving a plane horizontally) resemble the anisotropic manner in which the simulated networks are formed (endothelial tip cells move from left to right, in response to a prescribed chemoattractant field). Furthermore, the LTR filtration can determine the location of anastomosis events and the RTL filtration can determine the location of branching events (see also our response to comment 13 from reviewer 1 above).

   Action: We have added the following text to the "Sweeping plane descriptor vectors cluster simulations by parameter values" section:

   > *We suggest that the LTR and RTL plane sweeping filtrations produce the most interpretable clusterings because sweeping the plane horizontally resembles the way in which endothelial tip cells migrate from left to right in our simulated data.*
   >
   > *No single descriptor vector was able to robustly cluster simulations into biologically interpretable groups. We suggest that double descriptor vectors combining LTR and RTL descriptor vectors can robustly group simulations into biologically interpretable clusterings because the associated filtrations capture the ways in which vessel segments anastomose and branch, respectively.*

8. **Comment: Choosing the value of k: the elbow plot (S2 Fig) is not hugely convincing to justify k=5, and the decision appears to have been made somewhat post hoc based on the desired interpretation of the clusters. The silhouette score might provide more information, and should be relatively easy to calculate given that a standard distance metric is used by the k-means algorithm.**

   Response: We thank the reviewer for this helpful feedback. We agree that alternative methods could be used to determine the optimal number of clusterings. However, our goal for this study is not to develop a fully automated pipeline

and remove expert knowledge. Instead, we seek to develop a pipeline that we can interpret with expert knowledge to identify biologically interpretable groupings. We find that $k = 5$ provides a reasonable choice in terms of the elbow plot and biological interpretation.

Action: We have included the following text in the "Sweeping plane descriptor vectors cluster simulations by parameter values" section

> *We applied $k-$means clustering to all sweeping plane double descriptor vectors and computed the average OOS accuracy for a range of values of k, also known as an elbow plot (S2 Fig)... We note that more sophisticated descriptors could be used to justify this choice of k, including the silhouette score [36]. However, the focus of our study is to develop a pipeline that can be guided by expert knowledge, and we find that $k = 5$ appears well justified by the elbow plot and biological interpretation.*

Reviewer 3

1. <u>**Comment:**</u> **At the risk of sounding pedantic, the description of tumour-induced angiogenesis in Fig. 1 needs its language updated or simply make clear that some of the concepts are useful modelling abstractions but not necessarily biological mechanisms. For example, endothelial cells (ECs) don't "deposit" other ECs as they invade the avascular space. Since the formulation of the original A-C model, there have been substantial advances in understanding the dynamics of tip and stalk cells in sprouting angiogenesis. Furthermore, ECs don't branch into two cells, it is ECs along an existing sprout that initiate secondary sprouts and so on. It may be worth bringing the description of TAF (VEGF+bFGF) from the methods section to the main text.**

   <u>**Response:**</u> We totally agree with the reviewer that the A-C model is simplistic and that many, more recent models provide more detailed depictions of the multiple, biological mechanisms that regulate angiogenesis. We introduce these processes in the Introduction and revisit them in the "Conclusions and discussion" section where we discuss possible directions for future work (see our responses to comments 1 and 3 from Reviewer 1). We have also revised the legend to Figure 1 as per the reviewer's helpful suggestions.

   <u>Action 1:</u> We have included a more detailed description of the processes that regulate angiogenesis in the Introduction section, as well as a description of some of the shortcomings of the A-C model. The modified text in the Introduction is provided below:

   > *Here, we focus on* tumor-induced angiogenesis*, the process by which tumor cells stimulate the formation of new blood vessels from pre-existing vasculature [18]. When oxygen and nutrient levels within a population of tumor cells become too low to sustain a viable cell population, the tumor cells produce several growth factors, including vascular endothelial growth factor (VEGF), platelet-derived growth factor (PDGF) and basic fibroblast growth factor (bFGF), which diffuse through the surrounding tissue [12, 14, 40]. On contact with neighboring blood vessels, these tumor angiogenesis factors (or TAFs) increase the permeability of the vessel walls and loosen adhesive bonds between the endothelial cells that line the blood vessels [15]. The TAFs activate the endothelial cells to release proteases that degrade the basement membrane [30]. Activated tip endothelial cells then migrate away from the parent vessel and follow external cues, such as spatial gradients of VEGF and/or fibronectin [32, 45]. Stalk endothelial cells located behind the tip cells proliferate into the surrounding tissue matrix, causing the emerging vessel sprouts to elongate [41]. When tip endothelial cells from one sprout come into contact with tip or stalk endothelial cells from another sprout, the two endothelial cells may fuse or anastamose together, forming a new loop through which oxygen and nutrients may be transported [32]. In Fig 1 we present a schematic model of how these processes are coordinated.*
   >
   > *As the number of experimental and theoretical studies of angiogenesis has increased, so our knowledge of the mechanisms involved in its regulation has increased beyond the idealised description given above [28, 37]. For example, subcellular signalling involving the VEGF and delta-notch signalling pathways is known to influence*

whether an endothelial cell adopts a tip or stalk cell phenotype [6]. Further, immune cells, including macrophages, that are present in the tumor microenvironment, are known to release TAFs such as VEGF and matrix degrading proteases which facilitate tumor angiogenesis [28]. Mechanical stimuli also influence endothelial cell migration. For example, the fluid shear stress experienced by endothelial cells lining blood vessels drives their migration by causing them to form lamellipodia and focal adhesions at the front of the cell, in the flow direction, and to retract focal adhesions at the rear [26, 27].

Action 2: The legend to Fig 1 has been revised (see response to comment (3) from Reviewer 1):

2. **Comment: The argument of the paper goes that the standard quantitative descriptors in page 4 (inter-vessel spacing, number of branching points, tortuosity, etc) fail to capture higher dimensional information and facilitate network clustering. It would be very interesting to see how (little) effective these are at clustering the simulations similarly to Fig. 12. This comparison would provide quantitative evidence of the superior performance of the method proposed.**

   Response: We thank the reviewer for this suggestion. We agree that this figure should be included in the main text.

   Action: We have moved SI Figure 16 (original manuscript) into the main text (Fig 12 in the revised manuscript). References to the figure have also been updated.

3. **Comment: Fig. 12 and accompanying text: it would be interesting to also see the PIO$_0(K^{LTR})$ PIO$_0(K^{RTL})$ descriptors plotted on a 2D plane (via Principal Component Analysis for example) to get a sense of the distance between the clusters. Fig 13 indicates that classes 1 and 2 may be phenotypically closer than any other pair of classes.**

   Response: We thank the reviewer for this suggestion. We have included a new figure, S10 Fig, in the revised manuscript (shown below). The colors correspond to the groupings for each simulation. As expected, pairs from consecutive groups (*e.g.*, 1 & 2, 2 & 3) are closer to each other than pairs from non-adjacent groups.

   Action: We have included a new supplementary figure (S10 Fig) for this analysis, and have included the following text in the "Sweeping plane descriptor vectors cluster simulations by parameter values" section:

   We suggest that double descriptor vectors combining LTR and RTL descriptor vectors are able to robustly cluster simulations into biologically interpretable clusterings because they capture how vessel segments anastomose and branch, respectively. We performed principal components analysis to reduce the "PIO$_0(K^{LTR})$ & PIO$_0(K^{RTL})$" double descriptor vector to two principal components. The results presented in S10 Fig show that the simulations from each group are also clustered in this two-dimensional space.

4. **Comment: Page 18: my main criticism of the study is that the conclusion that the proposed descriptors cluster the networks in biologically meaningful way is highly dependent on the choice of k=5 in the**

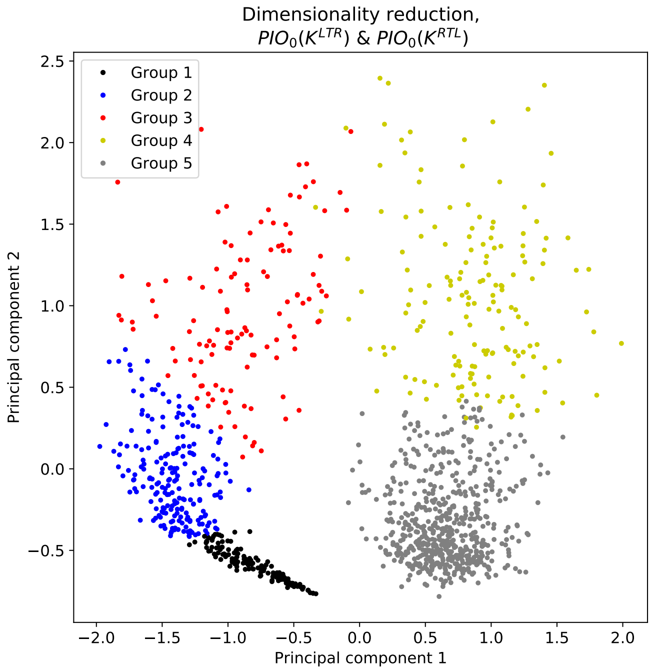

S10 Figure: Dimensionality reduction of the $\text{PIO}_0(K^{LTR})$ & $\text{PIO}_0(K^{RTL})$ double descriptor vector. We reduced the dimensionality of the $\text{PIO}_0(K^{LTR})$ & $\text{PIO}_0(K^{RTL})$ double descriptor vector down to two dimensions using principal components analysis and plot the reduced-dimension descriptor vector for each simulation. The color of each dot denotes the predicted grouping of each simulation from the k-means algorithm.

clustering analysis. This choice is justified in a very empirical way and I would suggest the authors to use the elbow method or a similar heuristic to argue about the optimal number of clusters in k-means based on the amount variance explained.

Response: We thank the reviewer for this helpful feedback. The conclusions certainly depend on the choice of $k = 5$ clusters, but this choice was guided by the elbow plot in S2 Fig of the revised manuscript. Further, we have included similar results for $k = 3$ and $k = 4$ in S8 Fig of the revised manuscript. While the results change slightly as k varies, we note that our goal is not to develop a fully automated pipeline and remove expert knowledge. Instead, we seek to develop a pipeline that we can interpret with expert knowledge to find biologically interpretable groupings. We find that $k = 5$ represents a compromise between choosing a small number of clusters and thoroughly partitioning the parameter space.

Action: We have included the following text in the "Sweeping plane descriptor vectors cluster simulations by parameter values" section

> *We applied $k-$means clustering to all sweeping plane double descriptor vectors and computed the average OOS accuracy for a range of values of k, also known as an elbow plot (S2 Fig)... We note that more sophisticated*

descriptors could have been used to justify this choice of k, including the silhouette score [36]. However, the focus of our study is to develop a pipeline that can be guided by expert knowledge, and we find that $k = 5$ appears well justified by the elbow plot and biological interpretation.

References

[1] H. Adams, T. Emerson, M. Kirby, R. Neville, C. Peterson, P. Shipman, S. Chepushtanova, E. Hanson, F. Motta, and L. Ziegelmeier. Persistence images: a stable vector representation of persistent homology. *Journal of Machine Learning Research*, 18(1):218–252, Jan. 2017.

[2] A. R. A. Anderson and M. A. J. Chaplain. Continuous and Discrete Mathematical Models of Tumor-induced Angiogenesis. *Bulletin of Mathematical Biology*, 60(5):857–899, Sept. 1998.

[3] J. W. Baish, T. Stylianopoulos, R. M. Lanning, W. S. Kamoun, D. Fukumura, L. L. Munn, and R. K. Jain. Scaling rules for diffusive drug delivery in tumor and normal tissues. *Proceedings of the National Academy of Sciences*, 108(5):1799–1803, Feb. 2011. ISBN: 9781018154107 Publisher: National Academy of Sciences Section: Physical Sciences.

[4] D. Balding and D. McElwain. A mathematical model of tumour-induced capillary growth. *Journal of Theoretical Biology*, 114(1):53–73, May 1985.

[5] P. Bendich, J. S. Marron, E. Miller, A. Pieloch, and S. Skwerer. Persistent Homology Analysis of Brain Artery Trees. *Annals of Applied Statistics*, 10(1):198–218, 2016.

[6] K. Bentley, C. A. Franco, A. Philippides, R. Blanco, M. Dierkes, V. Gebala, F. Stanchi, M. Jones, I. M. Aspalter, G. Cagna, S. Weström, L. Claesson-Welsh, D. Vestweber, and H. Gerhardt. The role of differential VE-cadherin dynamics in cell rearrangement during angiogenesis. *Nature Cell Biology*, 16(4):309–321, Apr. 2014.

[7] H. M. Byrne. Dissecting cancer through mathematics: from the cell to the animal model. *Nature Reviews Cancer*, 10(3):221–230, Mar. 2010.

[8] H. M. Byrne, H. A. Harrington, R. Muschel, G. Reinert, B. J. Stolz, and U. Tillmann. Topology characterises tumour vasculature. *Mathematics Today*, 55(5):206 – 210, 2019.

[9] H. Cai, X. Liu, J. Zheng, Y. Xue, J. Ma, Z. Li, Z. Xi, Z. Li, M. Bao, and Y. Liu. Long non-coding RNA taurine upregulated 1 enhances tumor-induced angiogenesis through inhibiting microRNA-299 in human glioblastoma. *Oncogene*, 36(3):318–331, Jan. 2017. Number: 3 Publisher: Nature Publishing Group.

[10] W. Chen, P. Xia, H. Wang, J. Tu, X. Liang, X. Zhang, and L. Li. The endothelial tip-stalk cell selection and shuffling during angiogenesis. *Journal of Cell Communication and Signaling*, 13(3):291–301, Sept. 2019.

[11] H. Edelsbrunner and J. Harer. Persistent homology–a survey. In J. E. Goodman, J. Pach, and R. Pollack, editors, *Contemporary Mathematics*, volume 453, pages 257–282. American Mathematical Society, Providence, Rhode Island, 2008.

[12] N. Ferrara. VEGF and the quest for tumour angiogenesis factors. *Nature Reviews Cancer*, 2(10):795–803, Oct. 2002. Number: 10 Publisher: Nature Publishing Group.

[13] A. A. Folarin, M. A. Konerding, J. Timonen, S. Nagl, and R. B. Pedley. Three-dimensional analysis of tumour vascular corrosion casts using stereoimaging and micro-computed tomography. *Microvascular Research*, 80(1):89–98, July 2010.

[14] J. Folkman. Tumor Angiogenesis. In G. Klein, S. Weinhouse, and A. Haddow, editors, *Advances in Cancer Research*, volume 19, pages 331–358. Academic Press, Jan. 1974.

[15] J. Folkman, E. Merler, C. Abernathy, and G. Williams. Isolation of a tumor factor responsible for angiogenesis. *The Journal of Experimental Medicine*, 133(2):275–288, Jan. 1971.

[16] K. Garside, R. Henderson, I. Makarenko, and C. Masoller. Topological data analysis of high resolution diabetic retinopathy images. *PLoS ONE*, 14(5):e0217413, May 2019. Publisher: Public Library of Science.

[17] J. A. Grogan, A. J. Connor, B. Markelc, R. J. Muschel, P. K. Maini, H. M. Byrne, and J. M. Pitt-Francis. Microvessel Chaste: An Open Library for Spatial Modeling of Vascularized Tissues. *Biophysical Journal*, 112(9):1767–1772, May 2017.

[18] M. K. Gupta and R.-Y. Qin. Mechanism and its regulation of tumor-induced angiogenesis. *World Journal of Gastroenterology*, 9(6):1144–1155, June 2003.

[19] M. Hadjicharalambous, P. A. Wijeratne, and V. Vavourakis. From tumour perfusion to drug delivery and clinical translation of in silico cancer models. *Methods*, 185:82–93, Jan. 2021.

[20] S. Hamis, G. G. Powathil, and M. A. Chaplain. Blackboard to Bedside: A Mathematical Modeling Bottom-Up Approach Toward Personalized Cancer Treatments. *JCO Clinical Cancer Informatics*, (3):1–11, Dec. 2019.

[21] L. Jakobsson, C. A. Franco, K. Bentley, R. T. Collins, B. Ponsioen, I. M. Aspalter, I. Rosewell, M. Busse, G. Thurston, A. Medvinsky, S. Schulte-Merker, and H. Gerhardt. Endothelial cells dynamically compete for the tip cell position during angiogenic sprouting. *Nature Cell Biology*, 12(10):943–953, Oct. 2010. Number: 10 Publisher: Nature Publishing Group.

[22] P. Kannan, W. W. Kretzschmar, H. Winter, D. Warren, R. Bates, P. D. Allen, N. Syed, B. Irving, B. W. Papiez, J. Kaeppler, B. Markelc, P. Kinchesh, S. Gilchrist, S. Smart, J. A. Schnabel, T. Maughan, A. L. Harris, R. J. Muschel, M. Partridge, R. A. Sharma, and V. Kersemans. Functional Parameters Derived from Magnetic Resonance Imaging Reflect Vascular Morphology in Preclinical Tumors and in Human Liver Metastases. *Clinical Cancer Research*, 24(19):4694–4704, Oct. 2018. Publisher: American Association for Cancer Research Section: Precision Medicine and Imaging.

[23] A. Karolak, D. A. Markov, L. J. McCawley, and K. A. Rejniak. Towards personalized computational oncology: from spatial models of tumour spheroids, to organoids, to tissues. *Journal of The Royal Society Interface*, 15(138):20170703, Jan. 2018. Publisher: Royal Society.

[24] M. A. Konerding, E. Fait, and A. Gaumann. 3D microvascular architecture of pre-cancerous lesions and invasive carcinomas of the colon. *British Journal of Cancer*, 84(10):1354–1362, May 2001. Number: 10 Publisher: Nature Publishing Group.

[25] M. A. Konerding, W. Malkusch, B. Klapthor, C. v. Ackern, E. Fait, S. A. Hill, C. Parkins, D. J. Chaplin, M. Presta, and J. Denekamp. Evidence for characteristic vascular patterns in solid tumours: quantitative studies using corrosion casts. *British Journal of Cancer*, 80(5):724–732, May 1999. Number: 5 Publisher: Nature Publishing Group.

[26] J.-L. Li and A. L. Harris. Notch signaling from tumor cells: A new mechanism of angiogenesis. *Cancer Cell*, 8(1):1–3, July 2005.

[27] S. Li, P. Butler, Y. Wang, Y. Hu, D. C. Han, S. Usami, J.-L. Guan, and S. Chien. The role of the dynamics of focal adhesion kinase in the mechanotaxis of endothelial cells. *Proceedings of the National Academy of Sciences*, 99(6):3546–3551, Mar. 2002. Publisher: National Academy of Sciences Section: Physical Sciences.

[28] R. Lugano, M. Ramachandran, and A. Dimberg. Tumor angiogenesis: causes, consequences, challenges and opportunities. *Cellular and Molecular Life Sciences*, 77(9):1745–1770, May 2020.

[29] N. V. Mantzaris, S. Webb, and H. G. Othmer. Mathematical modeling of tumor-induced angiogenesis. *Journal of Mathematical Biology*, 49(2):111–187, Aug. 2004.

[30] S. Menashi, H. Lu, C. Soria, and Y. Legrand. 2 Endothelial cell proteases: Physiological role and regulation. *BailliÃšre's Clinical Haematology*, 6(3):559–576, Sept. 1993.

[31] J. Metzcar, Y. Wang, R. Heiland, and P. Macklin. A Review of Cell-Based Computational Modeling in Cancer Biology. *JCO Clinical Cancer Informatics*, Feb. 2019. Publisher: American Society of Clinical Oncology.

[32] N. Paweletz and M. Knierim. Tumor-related angiogenesis. *Critical Reviews in Oncology/Hematology*, 9(3):197–242, Jan. 1989.

[33] S. M. Peirce. Computational and Mathematical Modeling of Angiogenesis. *Microcirculation*, 15(8):739–751, 2008. _eprint: https://onlinelibrary.wiley.com/doi/pdf/10.1080/10739680802220331.

[34] H. Perfahl, B. D. Hughes, T. Alarcón, P. K. Maini, M. C. Lloyd, M. Reuss, and H. M. Byrne. 3D hybrid modelling of vascular network formation. *Journal of Theoretical Biology*, 414:254–268, 2017.

[35] R. C. Rockne, A. Hawkins-Daarud, K. R. Swanson, J. P. Sluka, J. A. Glazier, P. Macklin, D. A. Hormuth, A. M. Jarrett, E. A. B. F. Lima, J. Tinsley Oden, G. Biros, T. E. Yankeelov, K. Curtius, I. Al Bakir, D. Wodarz, N. Komarova, L. Aparicio, M. Bordyuh, R. Rabadan, S. D. Finley, H. Enderling, J. Caudell, E. G. Moros, A. R. A. Anderson, R. A. Gatenby, A. Kaznatcheev, P. Jeavons, N. Krishnan, J. Pelesko, R. R. Wadhwa, N. Yoon, D. Nichol, A. Marusyk, M. Hinczewski, and J. G. Scott. The 2019 mathematical oncology roadmap. *Physical Biology*, 16(4):041005, June 2019.

[36] P. J. Rousseeuw. Silhouettes: A graphical aid to the interpretation and validation of cluster analysis. *Journal of Computational and Applied Mathematics*, 20:53–65, Nov. 1987.

[37] H. Saman, S. S. Raza, S. Uddin, and K. Rasul. Inducing Angiogenesis, a Key Step in Cancer Vascularization, and Treatment Approaches. *Cancers*, 12(5):1172, May 2020. Number: 5 Publisher: Multidisciplinary Digital Publishing Institute.

[38] M. Scianna, C. G. Bell, and L. Preziosi. A review of mathematical models for the formation of vascular networks. *Journal of Theoretical Biology*, 333:174–209, Sept. 2013.

[39] M. Sefidgar, M. Soltani, K. Raahemifar, M. Sadeghi, H. Bazmara, M. Bazargan, and M. Mousavi Naeenian. Numerical modeling of drug delivery in a dynamic solid tumor microvasculature. *Microvascular Research*, 99:43–56, May 2015.

[40] L. M. Sherwood, E. E. Parris, and J. Folkman. Tumor Angiogenesis: Therapeutic Implications. *New England Journal of Medicine*, 285(21):1182–1186, Nov. 1971.

[41] M. Sholley, G. Ferguson, H. Seibel, J. Montour, and J. Wilson. Mechanisms of neovascularization. Vascular sprouting can occur without proliferation of endothelial cells. *Laboratory Investigation; a Journal of Technical Methods and Pathology*, 51(6):624–634, Dec. 1984.

[42] D. Stepanova, H. M. Byrne, P. K. Maini, and T. AlarcÃ$^3n.Amultiscalemodelofcomplexendothelialcelldynamicsinearlyangiogene$[illegible]
$e1008055, Jan.2021. Publisher: PublicLibraryofScience.$

[43] B. J. Stolz, J. Kaeppler, B. Markelc, F. Mech, F. Lipsmeier, R. J. Muschel, H. M. Byrne, and H. A. Harrington. Multiscale topology characterises dynamic tumour vascular networks. *arXiv:2008.08667 [q-bio]*, 2020.

[44] B. Stolz-Pretzer. *Global and local persistent homology for the shape and classification of biological data.* http://purl.org/dc/dcmitype/Text, University of Oxford, Jan. 2019.

[45] V. P. Terranova, R. DiFlorio, R. M. Lyall, S. Hic, R. Friesel, and T. Maciag. Human endothelial cells are chemotactic to endothelial cell growth factor and heparin. *Journal of Cell Biology*, 101(6):2330–2334, Dec. 1985.

[46] V. Vavourakis, P. A. Wijeratne, R. Shipley, M. Loizidou, T. Stylianopoulos, and D. J. Hawkes. A Validated Multiscale In-Silico Model for Mechano-sensitive Tumour Angiogenesis and Growth. *PLoS Computational Biology*, 13(1):e1005259, Jan. 2017. Publisher: Public Library of Science.

[47] G. Vilanova, M. BurÃ©s, I. Colominas, and H. Gomez. Computational modelling suggests complex interactions between interstitial flow and tumour angiogenesis. *Journal of The Royal Society Interface*, 15(146):20180415, Sept. 2018.

[48] G. Vilanova, I. Colominas, and H. Gomez. A mathematical model of tumour angiogenesis: growth, regression and regrowth. *Journal of The Royal Society Interface*, 14(126):20160918, Jan. 2017. Publisher: Royal Society.
